# GEMReg: a spatio-temporal grayordinate ensemble modelling framework for predicting task activation maps from resting-state fMRI

**DOI:** 10.3389/fnins.2025.1716271

**Published:** 2025-12-03

**Authors:** Sasideep Pasumarthi, Satwik Bathula, Nitya Tiwari, Himanshu Padole

**Affiliations:** School of Electrical and Computer Sciences, Indian Institute of Technology, Bhubaneswar, India

**Keywords:** activation map prediction, functional MRI (fMRI), histogram, temporal feature extraction, time series regression

## Abstract

**Objective:**

Recent advances in neuroimaging have highlighted the growing utility of resting-state functional magnetic resonance imaging (rs-fMRI) as an alternative to task-based fMRI. In addition to being simpler, cost-effective, and time-efficient, rs-fMRI is particularly advantageous for non-compliant populations such as infants, elderly individuals, and patients with physical or cognitive impairments.

**Methods:**

Motivated by this, the present study introduces a novel Grayordinate Ensemble Modeling for Regression (GEMReg) framework for predicting task activation maps solely from rs-fMRI data, which, for the first time, leverages the rich temporal information of rs-fMRI for the task activation maps prediction. Specifically, the proposed approach uniquely formulates the task-activation map prediction as time series regression and exploits different temporal features and representations of the rs-fMRI for the same, including the proposed novel histogram-based features. Focusing on the individual characteristics of the grayordinates, 59412 individualized models (one per grayordinate) were trained by employing multiple univariate time series regressors. To optimize the prediction performance, a novel GEMReg framework is developed that selects the optimal feature-regressor combination for each grayordinate, exploiting the subtle variances in the individual grayordinate mapping. Furthermore, the temporal feature-based GEMReg is integrated with conventional functional connectivity maps-based spatial features, resulting in the spatio-temporal GEMReg, uniquely benefiting from both temporal and spatial features.

**Results and conclusion:**

Comparative analyses demonstrate that the proposed spatio-temporal GEMReg consistently outperforms existing methods across standard evaluation metrics, thereby establishing a new state-of-the-art for task activation map prediction using rs-fMRI.

## 1 Introduction

With the recent advances in neuroimaging and data analysis, along with traditional magnetic resonance imaging (MRI), the functional MRI (fMRI) has also found widespread applications, both in neuroscience research and clinical investigations. fMRI (Heeger and Ress, 2002) is a neuroimaging method that detects changes in blood flow and oxygenation in the brain to locate active areas during tasks or mental activities, leading to the neural activation maps. Depending on the mode of acquisition, fMRI is broadly categorized into two categories, viz. task-based fMRI (task-fMRI) (Barch et al., 2013), where the fMRI signal is recorded while a subject performs a certain predefined activity or task, and resting-state fMRI (rs-fMRI) (Lee et al., 2013), which measures spontaneous brain activity while at rest, performing no explicit task. Task-fMRI is widely used in cognitive neuroscience and psychiatric research for mapping brain functions related to specific stimuli or behaviors (Silva et al., 2018), such as language processing, motor control, or visual perception. In clinical settings, it assists in pre-surgical planning by identifying eloquent brain regions in patients with tumors or epilepsy (Lakhani et al., 2023), thus helping neurosurgeons avoid critical functional areas during resection. rs-fMRI, on the other hand, is extensively applied in understanding neurological and psychiatric disorders such as Alzheimer's disease, schizophrenia, autism spectrum disorders, and depression, offering insights into altered connectivity patterns and potential biomarkers for early diagnosis and treatment monitoring (Canario et al., 2021). Although with its high functional specificity, task-fMRI is more commonly used, it requires experiments to be performed to locate the brain regions that are activated during particular tasks, such as social, hand movement, language, gambling, etc. Specifically, the blood oxygen level dependent (BOLD) signal recorded during the task is compared with the baseline signal to determine the regions of the brain that are active while performing that task. This acquisition process of task-fMRI poses some limitations, including the need for task compliance, individual variability in performance, and the restriction to predefined cognitive domains. For localizing different functional regions of the brain, subjects must perform different tasks inside a scanner, making task fMRI acquisition a time-consuming and costly process. In addition, since the tasks must be performed with precision, they must be performed under expert supervision, posing another challenge. Apart from this, the design of an optimal, still reasonably simple task for localizing a particular brain region is always a challenge. In addition to these general challenges, the acquisition of task-fMRI data becomes even more challenging when dealing with noncompliant subjects having difficulties in task performance, e.g., infants, older subjects, paralyzed subjects, and subjects with physical or mental disabilities. All these challenges ultimately restrict the practical applicability of fMRI, making it a more research-oriented neuroimaging modality. The challenges restricting the wider applicability of the fMRI can thus be addressed to a large extent if the actual task performance required in task-fMRI can be avoided while still getting the corresponding task activation maps.

With this motivation, in recent times, several attempts have been made to obtain the task-activation maps using only the rs-fMRI data, which does not require any task performance, unlike the task-fMRI (Tavor et al., 2016; Cohen et al., 2020; Lacosse et al., 2021; Kocak, 2021; Ellis and Aizenberg, 2020; Tik et al., 2023; Tetereva et al., 2022; Ngo et al., 2022; Zheng et al., 2022). Tavor et al. (2016), in their seminal work, formulated the task activation map prediction problem as a regression problem where the outcome is the desired task activation map and predictors are chosen to be the resting state functional connectivity maps, to exploit the similarity between the resting state functional connectivity maps and the activation maps. Linear regression models were learned for the volumetric pixels (voxels) in every brain parcel for each subject, and the average of the learned coefficients over all the other subjects was used for predicting the activation map of the test subjects. This resulted in correlation values between 0.13 and 0.80 for actual vs predicted activation maps for different contrasts. Although this simple linear regression-based model performed reasonably well, its performance was limited by the inherent linearity assumption. To overcome this limitation, Cohen et al. (2020) proposed a similar model but used more sophisticated methods for regression, like feed-forward neural networks and random forest. This resulted in a correlation in the range of 0.54 to 0.804 for actual vs predicted activation maps for different contrasts. In Lacosse et al. (2021), the authors followed a similar approach and applied a ridge regression method to perform the regression. Their model yielded a correlation value of 0.582 when averaged over all contrast targets.

Apart from these voxel-based prediction models, various whole cortex-based prediction models have also been proposed, where the entire connectivity maps are treated as predictors or features, and the entire task activation map is obtained as the target or outcome variable (Kocak, 2021; Ellis and Aizenberg, 2020; Tik et al., 2023; Tetereva et al., 2022; Ngo et al., 2022; Kwon et al., 2025; Zheng et al., 2022). In Kocak (2021), the authors trained a convolutional neural network (CNN)-based model to learn a relationship between input functional connectivity maps and a target task activation map. This resulted in a maximum dice coefficient of 0.40. Ellis and Aizenberg (2020) opted for a similar CNN-based approach but used the structural connectivity-based maps as the features for a regression model. Tik et al. (2023) used a piecewise general linear model (GLM) to predict task-evoked brain activity from resting-state fMRI and evaluated its generalizability across datasets and populations. They achieved correlation values of 0.54 and 0.69 for emotion and social contrasts, respectively. Tetereva et al. (2022) demonstrated that integrating task-fMRI across multiple tasks with other MRI modalities using stacked Elastic Net significantly improves prediction of task activity, resulting in a correlation of 0.57. Recently, more advanced deep learning-based models such as SurfCNN (Ngo et al., 2022) have been proposed, which utilize surface-based convolutional neural networks to predict individual task activation patterns from resting-state functional connectivity features. These models effectively capture spatial and topological relationships on the cortical surface and have shown promising results, achieving an AUC of 0.3 and a DICE coefficient of 0.67 in predicting activation maps across various cognitive tasks. Kwon et al. (2025) proposed SwiFUN (Swin fMRI UNet Transformer) to predict task-evoked activation maps, which directly models 4D resting-state dynamics with a Swin-UNETR architecture and contrastive loss and validated its performance on the UK Biobank and ABCD resting-state fMRI. The model outperformed prior methods (e.g., BrainSurfCNN), with up to 27% improvement in predictive accuracy for certain contrasts (e.g., FACES-PLACES) and preserved individual differences useful for predicting traits like age, sex, and depressive symptoms. Similarly, Zheng et al. (2022) introduced a sparse ensemble learning framework that integrates predictions from multiple base models to enhance robustness and generalization in mapping resting-state features to task-evoked brain activity. Their approach demonstrated improved predictive performance, achieving an accuracy of over 70% across different task conditions.

However, in contrast to voxel-based models, the performance of these whole-cortex-based methods is often limited by their assumption of a global, one-size-fits-all model across the entire cortical surface. Each gray matter pixel (grayordinate) can exhibit distinct connectivity profiles and activation patterns depending on its location and cognitive relevance, and thus, the mapping between resting-state features and task-evoked responses is likely to vary substantially across the cortex. By applying a single model uniformly across all grayordinates, these methods fail to capture region-specific relationships and subtle inter-individual differences in brain function, which may lead to reduced predictive accuracy. Apart from these approach-specific limitations, almost all existing task activation map prediction models utilize the heuristically derived resting-state functional connectivity maps as the predictors or the features for regression. While these maps effectively encode functional relationships among brain regions, they do not capture the complex temporal dynamics of brain activity. Connectivity maps, by summarizing interactions such as correlations between regions, lose the important temporal information present in rs-fMRI signals, leading to the omission of transient, task-relevant activity patterns that can be crucial for accurate task activation map prediction. Time series-based features, on the other hand, retain the essential temporal information from the sequential rs-fMRI data, allowing the model to capture transient fluctuations and non-stationary interactions that are lost in static connectivity representations. Moreover, since connectivity maps are derived features, bypassing them enables the model to learn directly from the original spatiotemporal rs-fMRI signals. Moreover, the potential advantage of the temporal characterization is supported by recent advances in deep learning (Kwon et al., 2025; Madsen et al., 2025), where sequential modeling of time series has proven effective for capturing complex dynamics.

With this motivation, to overcome the limitations of the existing methods, a novel task-activation map prediction approach is presented here, which uniquely exploits the rich temporal information of the rs-fMRI data and also combines it with the spatial connectivity information. Specifically, the task-activation map prediction is first formulated as a time-series regression task wherein the rs-fMRI time series at each grayordinate is modeled as a predictor while the corresponding z-score is the target variable. To perform the regression task, apart from exploring the existing time series regressors, a novel histogram-based regression method is proposed, which exploits a unique set of features derived from diverse representations of the rs-fMRI. The predictions are improved further by developing a novel grayordinate ensemble modelling for regression (GEMReg), which integrates the optimal temporal features for each grayordinate, with the spatial features obtained using functional connectivity, resulting in 59412 distinct prediction models, individually optimized for each grayordinate. The main contributions of this work can be summarized as follows.

1. Introduced a novel perspective for task-activation map prediction using rs-fMRI that uniquely leverages the rich temporal information of rs-fMRI through time series regression.
2. Proposed a novel histogram-based approach for time series regression, uniquely exploiting the histogram-based features from different representations of time series.
3. Constructed a grayordinate-wise modeling framework comprising 59,412 independent optimized prediction models–one per grayordinate—uniquely capturing the subtle variations among different grayordinates.
4. Designed an ensemble-based task activation map prediction approach, GEMReg, by employing the best among different temporal feature-regression models for each grayordinate, facilitating a comprehensive integration of diverse predictive insights.
5. Developed a unified spatio-temporal GEMReg by integrating GEMReg-based temporal features with the functional connectivity-based spatial representations, yielding state-of-the-art prediction performance.

The remainder of this paper is organized as follows: Section II introduces the related work and fundamental concepts required to appreciate the work presented later. Section III describes the proposed task activation map prediction models in detail, followed by Section IV, which presents a thorough performance analysis with an ablation study. Section V concludes the paper with possible directions for future work.

## 2 Background and related work

This section begins with an overview of works on task activation map prediction using rs-fMRI, followed by an introduction to the basic concepts of activation maps and task contrasts in fMRI, and concludes with a brief description of the different time series feature extractors.

### 2.1 Related work on task activation map prediction

This subsection summarizes key studies that serve as the baseline task activation map prediction models and form the basis for comparison in our experimental evaluation. These works represent the most influential approaches to predicting task activation maps from resting-state fMRI (rs-fMRI) and provide context for understanding the performance of the proposed GEMReg framework. Each study varies in its modeling strategy—ranging from linear regression to multi-modal integration—and highlights different ways of linking resting-state features to task-evoked activations. Tavor et al. (2016), in their seminal work, formulated the task activation map prediction problem as a regression problem where the outcome is the desired task activation map and predictors are chosen to be the resting state functional connectivity maps, to exploit the similarity between the resting state functional connectivity maps and the activation maps. In particular, group principal component analysis (G-PCA) and group independent component analysis (G-ICA) were applied on the resting-state fMRI to generate 98 functional connectivity maps, 66 corresponding to the cortical region and the remaining 32 corresponding to the subcortical region. Furthermore, 9 structural MRI features were concatenated with the above functional connectivity features to generate a final 107-length vector that served as a feature for a general linear regression model (GLM). Regression models for volumetric pixels (voxels) were learned for each brain parcel in each subject, and the average of the learned coefficients in all other subjects was used to predict the activation map of the test subjects. This resulted in correlation values between 0.13 and 0.80 for actual vs predicted activation maps for different contrasts in Human Connectome Project (HCP) (Van Essen et al., 2013) dataset. Tik et al. (2023) followed a similar feature extraction strategy and employed PCA and ICA on the resting-state fMRI to generate 45 functional connectivity maps. However, in contrast to Tavor et al. (2016), instead of group PCA, Incremental PCA was applied, and group-ICA was carried out on both hemispheres, and not on each separately. The 45 resulting functional connectivity features were applied to a piecewise GLM, which predicted task-evoked brain activity from these features. The prediction performance of the model was evaluated using the HCP dataset, achieving correlation values of 0.54 and 0.69 for emotion and social contrasts, respectively. Tetereva et al. (2022) demonstrated that integrating task-fMRI across multiple tasks with other MRI modalities improves the prediction of task activity. Specifically, their study combined task-based functional MRI (tfMRI) from multiple cognitive tasks with non-task modalities, including structural MRI and resting-state functional connectivity from the HCP data. By comparing flat and stacked multimodal integration approaches across 16 machine learning configurations, the authors found that the stacked Elastic Net model—integrating all modalities–yielded the strongest predictive performance with the highest correlation of 0.57 (for the Working Memory task), surpassing models based on individual modalities.

In summary, although the simple linear regression-based models (Tavor et al., 2016; Tik et al., 2023) generally performed well, their performance is limited by the inherent linearity assumption. Though the more recent works like Tetereva et al. (2022) tried to address this by exploiting different regressors, including support vector regression (SVR), random forest (RF), XGBoost (XGB), and ElasticNet (ENet), the existing models often rely on a single global regression model applied uniformly across the cortex, overlooking the spatial heterogeneity and region-specific functional relationships of different gray matter coordinates. Moreover, these approaches predominantly use static resting-state functional connectivity maps as predictors, which fail to capture the rich temporal dynamics inherent in rs-fMRI signals. To address these limitations, the proposed GEMReg framework integrates both temporal and spatial information by formulating task activation prediction as a time-series regression problem, followed by its integration with spatial connectivity-based features. Further, by exploiting the optimal spatio-temporal feature-regressor pair, GEMReg constructs individualized models for each grayordinate, leading to more accurate and spatially resolved task activation predictions.

### 2.2 Activation maps and task contrasts

**Figure 1:**
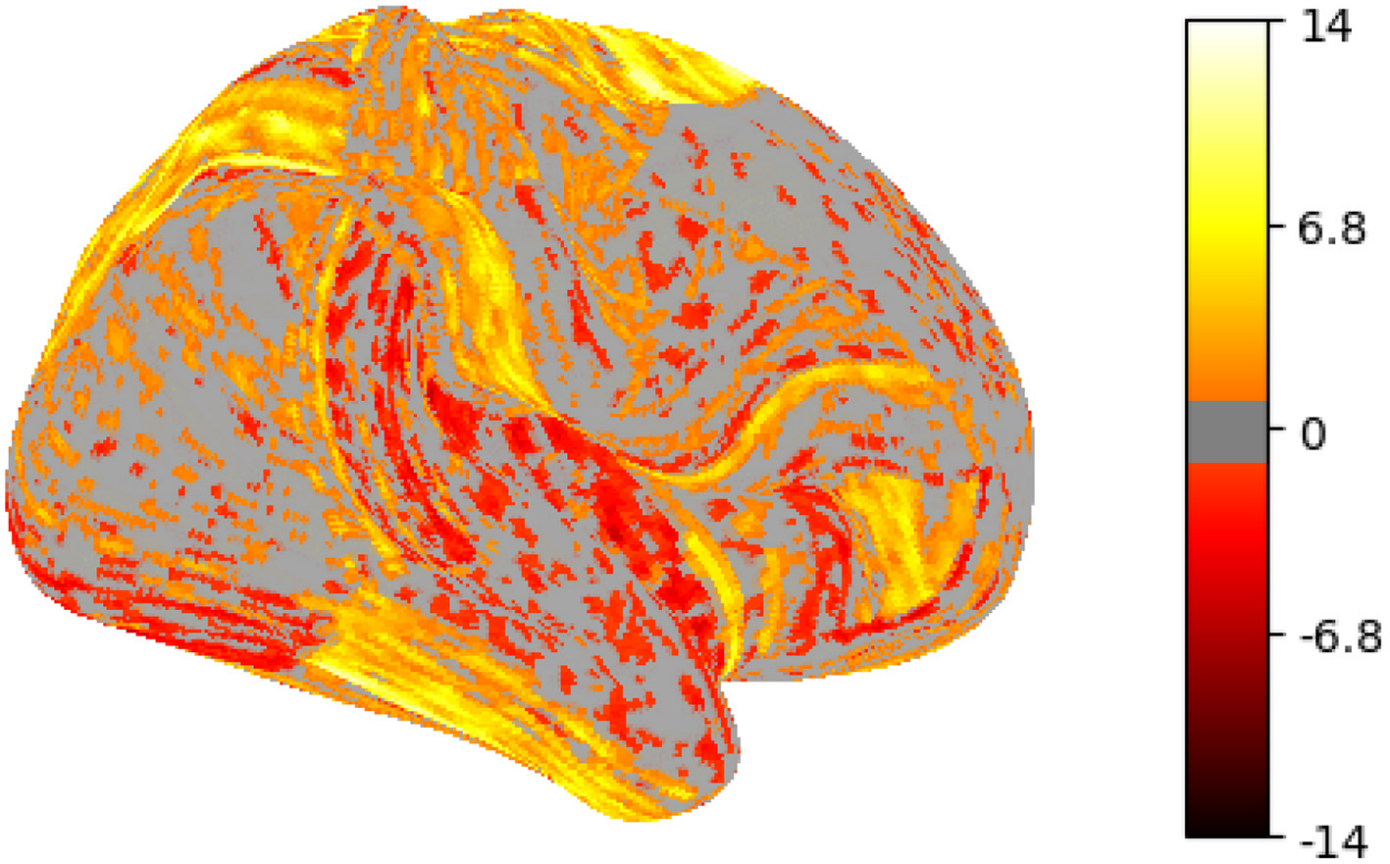
Example of an activation map obtained during EMOTION (cope1: FACES) contrast.

An activation map is a visual representation of brain activity that is obtained using techniques like fMRI. It shows areas of the brain that are more active during a particular task or in response to certain stimuli. Activation maps use color coding or intensity variations to indicate the strength of activity in different brain regions, helping researchers identify which parts of the brain are involved in specific cognitive processes or functions. Figure 1 shows a representative example of an activation map for the emotion task (cope1: FACES), highlighting the brain regions activated during the emotional task. Task activation maps are derived in task-fMRI by computing statistical contrasts between two conditions, i.e., task vs. baseline. These contrasts isolate brain activity specific to a particular cognitive function, and the resulting activation maps highlight regions with significantly different BOLD responses between the compared conditions.

The Human Connectome Project (HCP) (Van Essen et al., 2013) dataset utilized herein consists of fMRI data corresponding to a total of 86 contrasts from 7 tasks, viz., EMOTION, GAMBLING, LANGUAGE, MOTOR, RELATIONAL, SOCIAL, and WORKING MEMORY, with each task containing multiple contrasts. Of these 86 contrasts, the present study selected 7 contrasts randomly, one from each task, to validate the generalizability of the prediction models. Specifically, the contrasts considered in this study are: Emotion (cope1: FACES), Language (cope1: MATH), Relational (cope1: MATCH), Gambling (cope3: REWARD-PUNISH), Social (cope2: TOM), Working Memory (cope3: 2BK _PLACE), and Motor functions (cope3: LH). In Emotion, the selected contrast identifies brain regions activated by viewing fearful or neutral faces compared to control conditions, e.g., shape matching. In Language, the selected contrast identifies brain regions involved in mathematical processing, where participants perform arithmetic operations. In Relation, cope 1 identifies brain activity related to performing a relational matching task. In Gambling, cope3 highlights regions activated more strongly by winning compared to losing in a gambling task. In Social, cope2 identifies areas involved in understanding the intentions and beliefs of others by contrasting social interactions with non-social movements. In Working Memory, cope3 captures activation in a 2-back working memory task for places vs. baseline. In Motor, LH describes Left Hand Movement, highlighting brain areas involved in motor control. With this basic background of fMRI, the next subsection presents a brief overview of the time series feature extraction employed in the proposed work.

### 2.3 Time series feature extraction

The task activation map prediction models developed in this study incorporated a range of state-of-the-art time series feature extraction techniques, including convolution-based methods such as MRHydra (Tan et al., 2022), ROCKET (Dempster et al., 2020) and HYDRA (Dempster et al., 2023); interval-based approaches like DRCIF (Middlehurst et al., 2021) and RISE (Lines et al., 2018); and feature-based methods such as CATCH22 (Lubba et al., 2019) and SUMMARY (Guijo-Rubio et al., 2024). Among them, MrHydra, HYDRA, and ROCKET are convolution-based approaches designed to capture local and multiscale temporal patterns. Specifically, HYDRA is a dictionary-based algorithm that applies random convolutional kernels organized into multiple groups to obtain a feature representation from a time series. Depending on the number of kernels and groups, HYDRA typically produces between 8, 192 and 32, 768 features. Similarly, ROCKET uses 10, 000 convolutional kernels and two pooling operators to generate a 20, 000 length feature vector for each time series. MrHydra combines HYDRA and MultiRocket outputs, producing a concatenated feature vector. In contrast, interval-based methods such as DRCIF and RISE target transient dynamics within specific temporal segments. DRCIF extends CATCH22 with seven additional summary features, generating 29 features per interval across the *m*(*m*−1)/2 intervals, for time series length *m*. RISE randomly samples intervals, computing autocorrelation (ACF) and periodogram (PT) features, which are concatenated and classified using an ensemble of trees. As revealed through the ablation analysis, feature-based extractors—particularly CATCH22 and SUMMARY—consistently outperformed the others in this context. Accordingly, these two approaches are discussed in further detail below.

CATCH22—This feature extraction framework consists of the Canonical Time-series Characteristics pipeline, which computes 22 statistically significant features from raw time series data. These 22 features are derived from domains like temporal correlation, entropy, and distributional metrics by capturing the importance of the signal and reducing the dimensionality.

SUMMARY—In this feature extraction method, the SevenNumber SUMMARY transformer is employed to convert the input time series into summary statistics (Guijo-Rubio et al., 2024). The features extracted here include the mean, standard deviation, minimum, maximum, and the 0.25, 0.5, and 0.75 percentiles derived from the raw signal.

Equipped with the necessary background, the next section details the proposed task activation map prediction models.

## 3 Materials and methods

This section begins with a brief description of the dataset used herein and its preprocessing pipeline, followed by a detailed explanation of the proposed task-activation map prediction models.

### 3.1 Dataset and preprocessing

The entire analysis presented in this work utilizes the WU-Minn HCP dataset (Van Essen et al., 2013), one of the most extensively used publicly available neuroimaging datasets.

The project involves multimodal neuroimaging, behavioral, and genetic data acquisition, with a primary focus on young adults between the ages of 22 and 35 years, thereby minimizing age-related and pathological variability. In particular, the present study utilizes the S900 new release of the HCP, which contains data from approximately 336 subjects, each of whom had undergone rs-fMRI acquisition using a customized Siemens 3T Connectome Skyra scanner. For each subject, the dataset includes two rs-fMRI scans in the left-right (LR) and right-left (RL) phase-encoding directions, each with approximately 15 min of acquisition time, resulting in 1, 200 timepoints per run. To ensure data quality and consistency, the rs-fMRI data have been subjected to the HCP minimal preprocessing pipeline (Glasser et al., 2013), which includes spatial distortion correction, motion correction, intensity normalization, and surface-based registration. Specifically, the multimodal surface matching using all modalities (MSM-All) algorithm was applied for cross-subject alignment. Non-neuronal artifacts such as physiological noise (e.g., cardiac and respiratory signals), head motion, and scanner-related drift were removed using FMRIB's ICA-based X-noisefier (FIX), a robust automated denoising method that identifies and removes artifact-related independent components from the fMRI signal. Following preprocessing and denoising, the functional data were represented in CIFTI format as grayordinate time series, a unified data structure that captures both cortical surface vertices and subcortical volumetric voxels. The cortical data were mapped onto the 32*k*_*fsLR*_ surface mesh per hemisphere, while subcortical structures were defined in volumetric space. As a result, each subject's rs-fMRI time series is represented as a matrix of shape 91, 282 × 1, 200, where 91, 282 grayordinates span the cortex and subcortex, and 1,200 corresponds to the number of timepoints. Finally, to facilitate the design of the conventional spatial connectivity maps-based prediction model described next, the high-resolution grayordinate data were parcellated using HCP's multi-modal parcellation (MMP) 1.0 (Van Essen et al., 2013), which divides the brain into 379 distinct regions. Similar to all the earlier works (Kocak, 2021; Ellis and Aizenberg, 2020; Tik et al., 2023; Tetereva et al., 2022), as the present study is also interested in the prediction of cortical activity, the further analyses were restricted to 59, 412 grayordinates from 360 brain parcels that belong to the cortical region. The cerebral cortex data was particularly prioritized because it is often central to understanding higher-order cognitive functions, with significant implications for a broad range of neuropsychological applications (Lakhani et al., 2023; Canario et al., 2021). With this background, the following subsection provides a detailed explanation of the temporal feature-based task-activation map prediction model and its subsequent integration with spatial connectivity features, which together form our proposed spatio-temporal GEMReg model.

### 3.2 Task activation map prediction using temporal features

**Figure 2:**
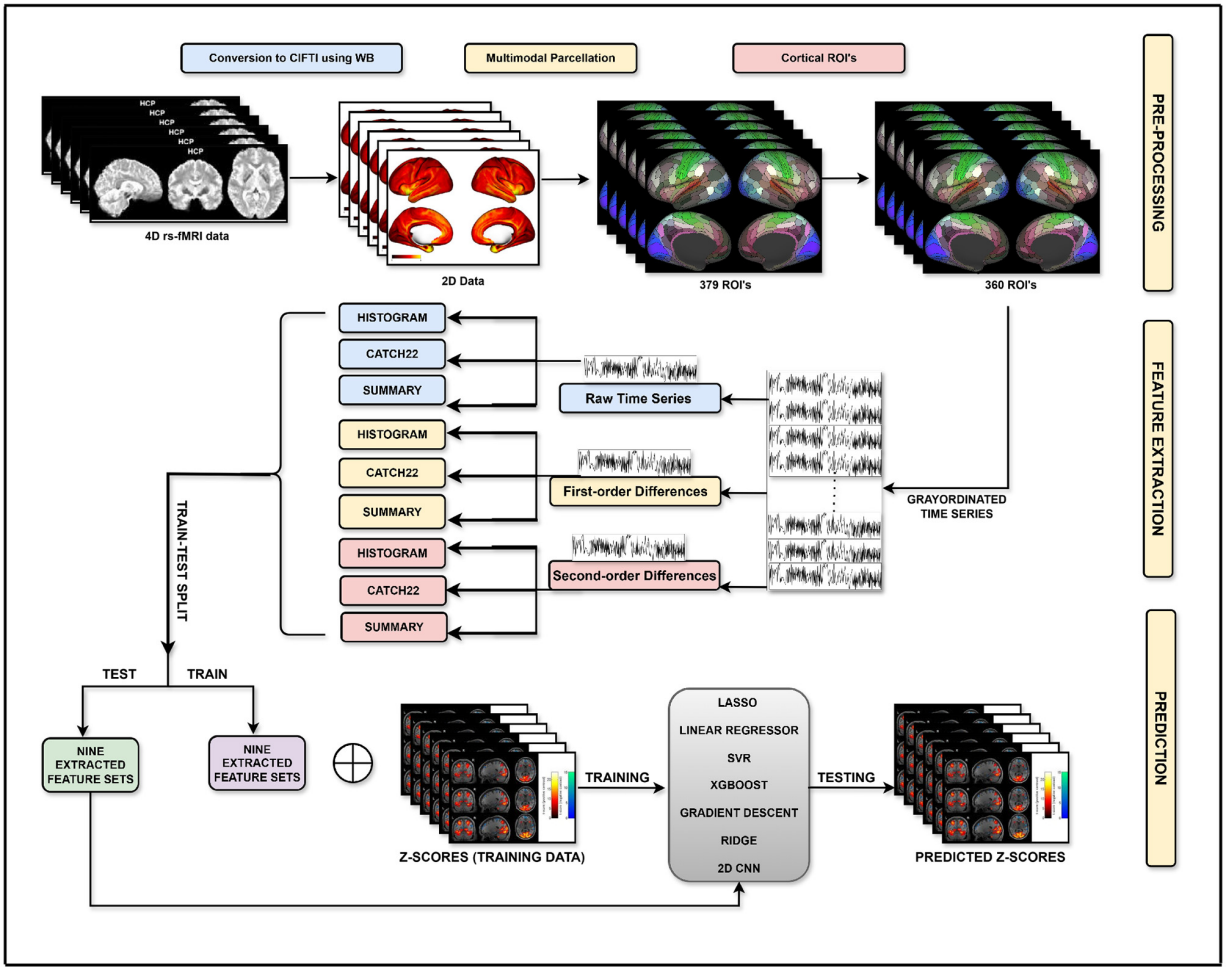
Pipeline for prediction of task activation maps using rs-fMRI time series regression.

As mentioned in Section 3.1, the preprocessing of the rs-fMRI for each subject results in data with dimensions of 91, 282 × 1, 200, where 91, 282 is the number of grayordinates and 1, 200 is the number of time points. When restricted to the cortex part of the brain, the data reduces to a size of 59, 412 × 1, 200, with 59, 412 corresponding to a number of grayordinates in the cortical region. With this data as input, the prediction model is expected to predict the task-activation map, which is a 59, 412 valued vector containing a z-score value at each cortical grayordinate. Now, as the input to the prediction model is essentially a set of 59, 412 rs-fMRI BOLD time series, here for the first time, we formulate the task-activation map prediction problem as a time series regression problem, to exploit the intrinsic temporal information of the rs-fMRI. Specifically, the task-activation map prediction task is uniquely formulated as 59, 412 univariate time series regression problems, where the prediction of a z-score at each of the 59, 412 grayordinates is obtained through its own tailored time series regression model trained using the corresponding rs-fMRI time series. Figure 2 depicts the overall pipeline of the proposed time-series regression-based activation map prediction approach. To validate the proposed approach, initially, the existing state-of-the-art time series regression methods, introduced in Section 2.3, are explored. Further, to improve upon it, a novel histogram-based time series regression method is proposed, and its utility in the present application is also validated.

#### 3.2.1 Task activation map prediction using CATCH22 and SUMMARY features

As discussed above, the proposed task-activation map prediction approach formulates the prediction task as 59, 412 univariate time series regression problems, where the prediction of a z-score at each of the 59, 412 grayordinates is obtained through its own tailored regression model trained using the corresponding 1, 200-length rs-fMRI time series. To train the time series regression models, the entire rs-fMRI data of 336 subjects was first divided into a training and a testing set in an 80:20 ratio, thus resulting in a training set of 268 samples for each grayordinate's prediction model. Following this, different time series feature extraction methods, as discussed in Section 2.3, were applied, generating a corresponding feature set for its z-score prediction. To further capture the temporal dynamics of the fMRI signal, the features were also computed from two additional representations of the rs-fMRI time series, i.e., first-order and second-order differences of the raw time series. These derivatives emphasize signal changes and accelerations or curvature, respectively, potentially unveiling transient or subtle temporal patterns not easily recognizable in the raw data.

As detailed in Section 4.2.2, among the various state-of-the-art time series features, CATCH 22 and SUMMARY were found to be the optimal in the present application. Both these features were computed for each representation, resulting in six feature sets: CATCH22, first-order CATCH22, second-order CATCH22, SUMMARY, first-order SUMMARY, and second-order SUMMARY. Subsequently, various standard regression models such as linear regression (LR), LASSO regression, random forest (RF), XGBoost, gradient boosting (GB), and support vector regression (SVR), were trained using each of these six feature sets to find the optimal combination of the feature set and a regressor. As detailed in Section 4.2, the SUMMARY features applied to the SVR performed the best, yielding correlation values in the range of 0.11 to 0.72 between the actual and the predicted activation maps. Although this validates the efficacy of the proposed time series regression-based task activation map prediction approach, to improve it further, we next propose a novel histogram-based time series regression method and verify its effectiveness in the present application.

#### 3.2.2 Task activation map prediction using proposed histogram-based features

As discussed above, the conventional summary statistics such as the mean, standard deviation, slope, and percentile measures (e.g., 0.25, 0.50, 0.75, and 1.00), coupled with the SVR, provide an impressive prediction performance, with correlation values up to 0.72. Inspired by this, here, for the first time, we propose to employ histograms as an alternative feature representation for rs-fMRI time series. Unlike the summary statistics, which yield only a small set of scalar descriptors, histograms capture the entire distribution of signal fluctuations within a time window, thereby preserving richer information about the underlying dynamics. This particularly helps in characterizing subtle changes in the signal that may be lost when using a single global measure.

However, since rs-fMRI signals can only be approximated as locally stationary, it is necessary to analyze them over shorter temporal windows, where the statistical characteristics of the signal, modeled by the histogram, can be assumed to remain unchanged, So, unlike the existing statistical summary features, which are essentially computed over the entire signal, the proposed histogram features were computed from smaller non-overlapping windows, over which the signal can be assumed to be stationary. As suggested by the prior work in the fMRI data analysis (Leonardi and Van De Ville, 2015; Preti et al., 2017), the assumption of stationarity, for the rs-fMRI signal, typically holds within windows of approximately 30–60 seconds [corresponding to 40–80 TRs (Repetition Time) in the HCP dataset, with TR = 0.72 s]. Motivated by this, each rs-fMRI time series of 1,200 timepoints, corresponding to a total duration ≈ 864 s, was first segmented into *N* non-overlapping segments. In this study, *N* was set to be 20, resulting in 60 timepoints per segment (≈43.2 s), thus respecting the assumption of stationarity. The proposed segmentation-based approach ensured that temporally local characteristics of the rs-fMRI are preserved, while still capturing the evolving signal dynamics. Following the segmentation, for each segment, a histogram with 50 bins was computed, yielding a segment-wise histogram of size 20 × 50 per subject, which essentially summarized the local distributional properties of the rs-fMRI signal while retaining temporal variability across segments. A similar exercise was repeated for the first-order and second-order differences of the raw time series. Overall, the proposed approach is analogous to computing time-varying summary statistics but with substantially greater descriptive power.

**Figure 3:**
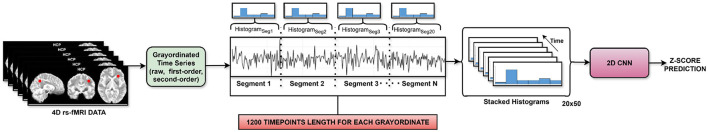
Pipeline for prediction of task activation maps from rs-fMRI using the proposed histogram-based time series features.

The resulting histogram matrices were then provided as input to a two-dimensional convolutional neural network (2D CNN) for the activation map prediction, as shown in Figure 3. Each input matrix had a size of 20 × 50, corresponding to 20 temporal segments and 50 histogram bins, and was treated as a single-channel two-dimensional image. As detailed in Section 4, among the different CNN architectures, the following architecture (referred to as CNN1) was found to be the optimal and hence was employed in all further analysis:

- **First convolutional layer:** 16 filters of size 3 × 3 with padding of 1, followed by ReLU activation.
- **Second convolutional layer:** 32 filters of size 3 × 3 with ReLU activation.
- **Adaptive average pooling layer:** Reduces the feature map to a fixed output size of 5 × 10 per channel.
- **Flattening:** Converts pooled feature maps into a single vector.
- **Fully connected layers:** The first layer contains 128 hidden units with ReLU activation; the second layer produces a single scalar output corresponding to the predicted activation value.

The network was trained in a regression setting using mean squared error (MSE) as the loss function. The results, as detailed in Table 1, demonstrate that the proposed histogram-based feature extraction outperforms existing methods, validating its applicability for predicting task activation maps from rs-fMRI data. Building on this background, we further analyzed grayordinate-to-grayordinate correlations for each feature set and identified the optimal temporal feature-based regression, which is explained in detail in the subsequent section.

**Table 1:** Performance metrics for HISTOGRAM-based features across all task contrasts.

**Contrast**	**Features**	***r***	**DICE**	**AUC**
RELATIONAL	HIST	0.710	0.7300	0.8100
	1st HIST	0.7111	0.7300	0.8100
	2nd HIST	0.712	0.7345	0.8000
EMOTION	HIST	0.5901	0.6514	0.7147
	1st HIST	0.5899	0.6516	0.7147
	2nd HIST	0.5932	0.6516	0.7148
LANGUAGE	HIST	0.4701	0.6333	0.6902
	1st HIST	0.4699	0.6333	0.6902
	2nd HIST	0.4721	0.6332	0.6902
GAMBLING	HIST	0.1109	0.5283	0.5403
	1st HIST	0.0899	0.5288	0.5409
	2nd HIST	0.1120	0.5285	0.5406
SOCIAL	HIST	0.7210	0.7468	0.8249
	1st HIST	0.7209	0.7467	0.8248
	2nd HIST	0.7211	0.7467	0.8249
WM	HIST	0.6662	0.7039	0.7767
	1st HIST	0.6601	0.7039	0.7767
	2nd HIST	0.6671	0.7039	0.7767
MOTOR	HIST	0.4998	0.6056	0.6556
	1st HIST	0.4903	0.6056	0.6556
	2nd HIST	0.5020	0.6057	0.6558

Here, HIST refers to HISTOGRAM.

### 3.3 Task activation map prediction using optimal temporal features: GEMReg

**Figure 4:**
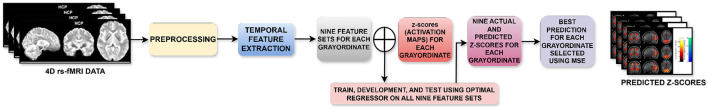
Pipeline for prediction of task activation maps using GEMReg with temporal features.

The prediction models proposed in the previous sections adopt a global modeling approach, wherein a single feature set is used uniformly across all grayordinates. However, since each grayordinate exhibits unique temporal dynamics and responds differently to various feature representations, such an approach may overlook important grayordinate-specific signal characteristics. To address this, a detailed evaluation of task-activation map prediction performance was conducted for each of the 59, 412 grayordinates using different regressors with nine distinct feature sets: HISTOGRAM, first-order HISTOGRAM, second-order HISTOGRAM, CATCH22, first-order CATCH22, second-order CATCH22, SUMMARY, first-order SUMMARY, and second-order SUMMARY, to determine the optimal feature-regressor combination for each grayordinate. Specifically, the evaluation dataset, consisting of the remaining 68 subjects, was divided into 34 subjects for development and 34 for the final testing. For every subject in the development dataset, four performance metrics—correlation coefficient (*r*), *r*^2^ score, mean absolute error (MAE), and mean squared error (MSE)—were computed for every grayordiante by applying each of the nine feature sets to different standard regressors, viz. LR, LASSO, SVR, Ridge, XGBoost, GB, and CNN1. As explained in detail in the ablation study, the feature-regressor combination yielding the lowest MSE across the development dataset was selected as the optimal for that grayordinate, resulting in superior overall activation map prediction. This localized feature-regressor selection strategy forms the foundation of our proposed task activation map prediction framework, GEMReg (Grayordinate Ensemble Modelling for Regression), as summarized in Figure 4. GEMReg performs ensemble modeling at the grayordinate level by dynamically selecting the most suitable feature-regressor for each grayordinate, rather than enforcing a one-size-fits-all model. The detailed mathematical framework of GEMReg is presented below.

#### Mathematical framework of GEMReg

Let *N* be the number of subjects, *G* the number of grayordinates, and *S* = 9 the number of feature sets extracted per grayordinate. Each feature set *s*∈{1, …, 9} yields a feature vector ${\text{x}}_{n,g}^{\left(s\right)}\in {\mathbb{R}}^{d_{s}}$ for subject *n* and grayordinate *g*, where *d*_*s*_ denotes the feature dimensionality of the *s*-th set. The corresponding target value (task activation z-score) is denoted by *y*_*n, g*_∈ℝ.

For each grayordinate *g* and feature set *s*, a separate regression model $f_{g}^{\left(s\right)}$ is trained using the training data ${\left\{\left({\text{x}}_{n,g}^{\left(s\right)},y_{n,g}\right)\right\}}_{n=1}^{N}$ by minimizing a regularized loss:


$$\begin{array}{l} f_{g}^{\left(s\right)}=\arg\underset{f\in H}{\min}L\left(f\left({\text{X}}_{g}^{\left(s\right)}\right),{\text{y}}_{g}\right) \end{array}$$ (1)

where H is the chosen model class (e.g., SVR, XGBoost, Ridge), L is the loss function (typically mean squared error).

In a held-out test set, for each grayordinate *g* and feature set *s*, its optimal regressor model $f_{g}^{\left(s\right)}$ obtained from (1), is used to generate predictions ${ŷ}_{n,g}^{\left(s\right)}$ as follows:


$$\begin{array}{l} {ŷ}_{n,g}^{\left(s\right)}=f_{g}^{\left(s\right)}\left({\text{x}}_{n,g}^{\left(s\right)}\right) \end{array}$$ (2)

Subsequently, mean squared error (MSE) across test subjects is computed for each feature set and grayordinate:


$$\begin{array}{l} {\text{MSE}}_{g}^{\left(s\right)}=\frac{1}{N_{\text{test}}}\overset{N_{\text{test}}}{\underset{n=1}{\sum }}{\left(y_{n,g}-{ŷ}_{n,g}^{\left(s\right)}\right)}^{2} \end{array}$$ (3)

The best-performing feature set for each grayordinate is then selected as:


$$\begin{array}{l} s_{g}^{*}=\arg\underset{s}{\min}{\text{MSE}}_{g}^{\left(s\right)} \end{array}$$ (4)

Accordingly, the final predicted activation z-score for subject *n* and grayordinate *g* is:


$$\begin{array}{l} {ŷ}_{n,g}={ŷ}_{n,g}^{\left(s_{g}^{*}\right)} \end{array}$$ (5)

The complete predicted task activation map for subject *n* is:


$$\begin{array}{l} {\hat{Y}}_{n}={\left[{ŷ}_{n,1},{ŷ}_{n,2},\ldots ,{ŷ}_{n,G}\right]}^{⊤}\in {\mathbb{R}}^{G} \end{array}$$ (6)

As detailed in Section 4.2, compared to the best-performing single global feature set, which achieved correlation values of 0.712, 0.5932, 0.4721, 0.6671, 0.1120, 0.502, 0.7211 for different contrasts, GEMReg consistently performed better with superior correlation values of 0.7201, 0.6042, 0.4746, 0.6560, 0.1430, 0.5029, 0.7258. This improvement validates our hypothesis that leveraging diverse temporal features on a per-grayordinate basis can better capture the intrinsic variability in brain signals. By exploiting grayordinate-specific feature-model combinations, GEMReg adapts to local signal dynamics and enhances the overall task-activation map prediction performance. Having optimized the time-series regression-based framework using GEMReg, next, we describe a spatial connectivity map-based prediction model, which is then integrated with GEMReg to construct spatio-temporal GEMReg.

### 3.4 Task activation map prediction using functional connectivity maps

This subsection introduces a conventional functional connectivity maps-based task activation map prediction approach where the spatial functional connectivity maps derived from rs-fMRI are used as predictors, and the z-scores of the activation maps are the target variables of the regression models. Inspired by the seminal work of Tavor et al. (2016), the same feature extraction process is followed, which essentially involves the generation of functional connectivity maps from the rs-fMRI data of 336 subjects, each with a data shape of 91, 282 × 1, 200. Incremental Principal Component Analysis (IPCA) was applied to the group data, resulting in reduced-dimensional data with a size of 91, 282 × 1, 000, followed by an independent component analysis with 40 components. Subsequently, functional connectivity maps were constructed using each of these 40 components in both hemispheres, thus resulting in a total of 80 functional connectivity maps. Among these, 31 functional connectivity maps from each hemisphere, which were symmetric between the left and right hemispheres, were selected as features for regression, yielding 62 cortical features. Similarly, for the sub-cortex region, the feature extraction method detailed in Tavor et al. (2016) resulted in 32 sub-cortical features. Combining cortical and sub-cortical features, the final dataset had a shape of 59, 412 × 94, where 59, 412 corresponds to the number of grayordinates in the cortical region, and 94 indicates the total number of features associated with each grayordinate.

Finally, the extracted features were provided as input to a GLM to predict the task-activation maps. Specifically, the brain was divided into 50 non-overlapping regions of interest (ROIs) derived through group ICA and a winner-takes-all parcellation applied to the ICA maps. For each subject, task activation values were predicted using parcel-wise linear regression models trained on features derived from resting-state fMRI. The cortex was parcellated into regions, and a separate regression model was fit within each parcel using only the grayordinates belonging to that parcel. To ensure inter-subject generalization, a leave-one-subject-out strategy was employed: for each test subject, the regression coefficients for each parcel were estimated by averaging the models trained on the remaining subjects. These averaged coefficients were then applied to the test subject's features within each parcel to generate the predicted task activation map.

Although this conventional approach performed reasonably well with correlation values between 0.087 to 0.680, its performance is fundamentally limited by the fact that it exploits only the spatial connectivity information from the rs-fMRI, neglecting the potentially useful rich temporal information in the rs-fMRI time series. With this motivation, we next propose a spatio-temporal GEMReg that uniquely combines the spatial connectivity maps-based features extracted herein with the temporal features of GEMReg for the enhanced task-activation map prediction.

### 3.5 Task activation map prediction using optimal spatio-temporal features: spatio-temporal GEMReg

**Figure 5:**
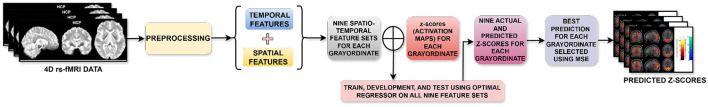
Pipeline for prediction of task activation maps using GEMReg with spatio-temporal features.

As discussed earlier, while the proposed multi-feature time series-based model, GEMReg, demonstrated state-of-the-art performance in predicting task activation maps, to improve the prediction performance further, we explored the integration of both spatial and temporal features into a unified modeling framework. Temporal features capture the dynamic properties of the fMRI signal, whereas spatial features reflect anatomical or functional connectivity patterns across grayordinates. Their integration provides a more comprehensive and complementary representation of brain activity, potentially enabling more robust modeling. To achieve this, the spatial connectivity maps-based features, detailed in Section 3.4, were concatenated with each of the nine temporal feature sets, i.e., HISTOGRAM, first- and second-order HISTOGRAM; CATCH22, first- and second-order CATCH22; and SUMMARY, first- and second-order SUMMARY. This resulted in nine distinct spatio-temporal feature sets, each representing a different combination of temporal dynamics and spatial context for every grayordinate. Following this, the proposed GEMReg framework was extended to operate over these spatio-temporal combinations. Specifically, for each grayordinate, GEMReg evaluated all nine spatio-temporal feature sets using different standard regressors, viz. LR, LASSO, SVR, Ridge, XGBoost, GB, and CNN1, across the development dataset and selected the optimal feature-regressor combination based on performance metrics such as MSE, correlation, *r*^2^ score, and MAE, as summarized in Figure 5. As detailed in the ablation study, among the four performance metrics, the lowest MSE determined the optimal feature-regressor. This spatio-temporal GEMReg framework ensured that the proposed prediction model for each grayordinate is informed not just by its temporal characteristics but also by spatial connectivity patterns, tailored dynamically via ensemble selection, potentially leading to improved performance. The detailed performance analysis presented in the next section demonstrates that the spatio-temporal GEMReg outperforms all the existing and our earlier proposed prediction models across all standard metrics, underscoring the benefit of exploiting local temporal variations alongside global spatial context.

## 4 Performance analysis

This section provides a detailed performance analysis of the task activation map prediction models proposed hitherto, followed by a comparison with the existing prediction methods. Subsequently, a thorough ablation study, conducted to find the optimal feature sets and regressors, and hence the optimal prediction models, is also presented. All the experiments herein were performed on rs-fMRI of 336 subjects from the HCP dataset, as detailed in Section 3.1. 80% of the total data was used for training the models, while the remaining 20% was used for development and testing purposes. The average performance of the prediction models over the test data was quantified using several metrics, including the Pearson correlation coefficient (*r*), coefficient of determination (*r*^2^), mean absolute error (MAE), the mean squared error (MSE), the area under the ROC curve (AUC) and the DICE coefficient, In our work, although the task is formulated as regression, we additionally report Dice and AUC scores in line with prior literature. Since both Dice and AUC require binary class labels, median thresholding is applied to the continuous values of both predicted and actual maps. This converts each column into a binary partition (above-median vs. below-median) (Tavor et al., 2016), with approximately equal numbers of positive and negative cases. Such thresholding is commonly used in neuroimaging studies to evaluate spatial overlap and discriminative ability, making our results directly comparable with existing work that reports Dice and AUC for activation maps, as defined below:

- **Pearson correlation coefficient (r)**: Measures the linear relationship between predicted and actual values.

$$\begin{array}{l} r=\frac{\sum_{i=1}^{n}\left(y_{i}-ȳ\right)\left({ŷ}_{i}-\bar{ŷ}\right)}{\sqrt{\sum_{i=1}^{n}{\left(y_{i}-ȳ\right)}^{2}}\sqrt{\sum_{i=1}^{n}{\left({ŷ}_{i}-\bar{ŷ}\right)}^{2}}} \end{array}$$ (7)

where *y*_*i*_ and ŷ_*i*_ are the actual and predicted values, respectively, and ȳ and $\bar{ŷ}$ are their respective means.

- **Coefficient of determination (*r*^2^)**: Represents the proportion of variance explained by the model.

$$\begin{array}{l} r^{2}=1-\frac{\sum_{i=1}^{n}{\left(y_{i}-{ŷ}_{i}\right)}^{2}}{\sum_{i=1}^{n}{\left(y_{i}-ȳ\right)}^{2}} \end{array}$$ (8)

- **Mean absolute error (MAE)**: Represents the average of the absolute differences between predicted and actual values.

$$\begin{array}{l} \text{MAE}=\frac{1}{n}\overset{n}{\underset{i=1}{\sum }}|y_{i}-{ŷ}_{i}| \end{array}$$ (9)

- **Mean squared error (MSE)**: Measures the average of the squares of the errors.

$$\begin{array}{l} \text{MSE}=\frac{1}{n}\overset{n}{\underset{i=1}{\sum }}{\left(y_{i}-{ŷ}_{i}\right)}^{2} \end{array}$$ (10)

- **Area under the curve (AUC)**: Represents the area under the Receiver Operating Characteristic (ROC) curve and is used to evaluate binary classification performance.
- **DICE coefficient**: Measures the spatial overlap between two binary volumes, often used for evaluating similarity in segmentation tasks.

$$\begin{array}{l} \text{DICE}=\frac{2|A\cap B|}{\left|A|+|B\right|} \end{array}$$ (11)

where *A* is the predicted set and *B* is the ground truth set.

### 4.1 Experimental results and discussion

This subsection presents the detailed performance analysis of the proposed task-activation map prediction models using three different feature configurations: (i) proposed histogram-based features, (ii) optimally selected temporal features, and (iii) optimally selected spatio-temporal features. Starting with the histogram-based features, CNN1 was employed to predict the *z*-scores across all task contrasts using this feature type. The resulting performance metrics for each contrast, described in Section 2.2, are reported in Table 1. In addition to CNN1, other CNN architectures were also tried, the performance of which is detailed in the ablation study in Section 4.2.1.

To evaluate the effectiveness of individual temporal feature sets, an ablation study was conducted, wherein the prediction performance of different feature extraction methods, mentioned in Section 4.2.2, was compared. The results of this ablation study, indicated CATCH22, SUMMARY, and the proposed HISTOGRAM-based features to be the top-performing features among all. So, for each of these three, further features were extracted from three different representations of the signal, viz. the raw, first-order difference, and second-order difference, resulting in a total of nine temporal feature variants. Subsequently, each of these nine feature sets was subjected to a range of regression algorithms used earlier, the results of which are summarized in the ablation study. As can be observed therein, SVR consistently outperformed other regressors for four out of the six existing temporal feature sets. However, the first- and second-order variants of the CATCH22 feature set exhibited better performance when modeled using LASSO Regression. Based on this analysis, SVR and LASSO were selected as the primary regressors for their respective feature sets. For the raw histogram, first-order histogram, and second-order histogram features, CNN1 consistently performed better compared to other CNN architectures, and hence was used in all the further regression analysis. To construct an optimal, grayordinate-wise temporal representation, different performance metrics, such as MSE, MAE, *r*^2^ score, and correlation, were computed for each of the nine feature sets at every grayordinate. The feature set yielding the best metric for a given grayordinate was selected as the optimal representation for that location. This procedure was repeated across all 59, 412 grayordinates. The corresponding performance metrics obtained from this optimal combination approach are presented in the ablation study. The results indicate that the optimally selected feature configuration leads to improved prediction performance across all contrasts, with MSE serving as the most reliable metric for determining grayordinate-wise feature set assignment. A summary of these results, with the optimal MSE-driven feature-regressor selection, is provided in Table 2.

**Table 2:** Performance metrics for optimally selected temporal features across all task contrasts.

**Contrasts**	***r***	**DICE**	**AUC**
RELATIONAL	0.7201	0.7317	0.8101
EMOTION	0.6042	0.6590	0.7253
LANGUAGE	0.4746	0.6309	0.7001
GAMBLING	0.1430	0.5387	0.5553
SOCIAL	0.7258	0.7329	0.8091
WM	0.6560	0.7011	0.7660
MOTOR	0.5029	0.6012	0.6601

For the spatio-temporal features, the analysis was extended by concatenating each of the nine temporal feature sets with connectivity maps-based spatial features obtained in Section 3.4, to create the combined features. Similar to earlier analysis, these combined spatio-temporal features were again provided to all seven aforementioned regression methods. Among these, LASSO exhibited superior performance for the three CATCH22-derived feature sets, while the three feature sets–corresponding to the Summary-based features—showed better results when modeled using SVR. For the histogram-based features, CNN1 yielded better results. The detailed performance metrics for all combinations of spatio-temporal features and regressors are presented in the ablation study. Following the same approach used for the temporal-only analysis, a grayordinate-wise performance analysis was conducted to identify the optimal spatio-temporal feature set for each of the 59,412 grayordinates. the results of which are reported in the ablation study. Among all evaluation metrics considered, i.e., correlation, *r*^2^ score, MSE, and MAE, MSE again emerged as the most reliable indicator of performance and was therefore used for guiding optimal feature assignment. The summary of performance metrics obtained using the optimal spatio-temporal features is provided in the ablation study.

A comparative analysis of the results in Tables 2, 3 reveals that incorporating spatio-temporal features leads to improved Pearson correlations, Dice coefficients, and AUC scores compared to temporal features alone, across all seven contrasts. These uniform improvements demonstrate that spatial context provides valuable complementary information, particularly for tasks with distributed or variable activation patterns, while offering limited benefit for highly localized tasks such as MOTOR. Consequently, spatio-temporal GEMReg becomes the natural choice for the subject-level prediction of task-evoked brain activity.

**Table 3:** Performance metrics for optimally selected spatio-temporal features across all task contrasts.

**Contrasts**	***r***	**DICE**	**AUC**
RELATIONAL	0.7273	0.7321	0.8109
EMOTION	0.6170	0.6599	0.7301
LANGUAGE	0.4858	0.6369	0.7101
GAMBLING	0.1510	0.5406	0.5648
SOCIAL	0.7299	0.7427	0.8101
WM	0.6721	0.7102	0.7841
MOTOR	0.5103	0.6121	0.6652

**Figure 6:**
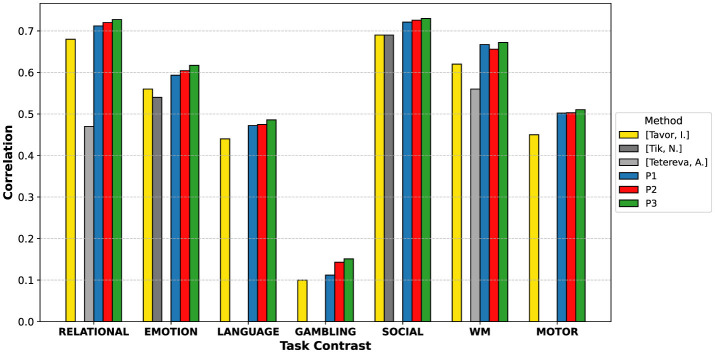
Comparison of task activation map prediction performance across methods and task contrasts (Tavor et al., 2016; Tik et al., 2023; Tetereva et al., 2022). P1, P2, and P3 represent the proposed histogram-based, optimal temporal feature-based, and optimal spatio-temporal feature-based models, respectively. The missing bars in comparisons indicate the unavailability of those results.

After a thorough evaluation presented above, Figure 6 summarizes the performance of the proposed task activation map prediction models in comparison with existing state-of-the-art prediction methods across all seven task contrasts: RELATIONAL, EMOTION, LANGUAGE, GAMBLING, SOCIAL, WORKING MEMORY (WM), and MOTOR. The rows labeled P1, P2, and P3 correspond to our proposed models based on (i) histogram-based regression, (ii) Temporal-based GEMReg, and (iii) the spatio-temporal-based GEMReg, respectively. From the comparison, it is evident that the proposed spatio-temporal GEMReg consistently outperforms all the existing methods as well as our other proposed models across all task contrasts, including more challenging contrasts like GAMBLING and LANGUAGE. The progression from P1 to P2 to P3 also demonstrates the incremental gains achieved by moving from fixed temporal features to per-grayordinate optimized temporal modeling, and ultimately to the integration of both spatial and temporal information. The state-of-the-art performance of the spatio-temporal GEMReg across all contrasts essentially validates our core hypothesis that grayordinate-wise modeling using diverse spatio-temporal features, coupled with ensemble-based feature selection, leads to more accurate and generalizable task activation map prediction. The improvement is particularly significant given the simplicity, cost-effectiveness, and accessibility of rs-fMRI data over task-based acquisitions.

Having validated the prediction performance of the proposed models through different quantitative metrics, we next provide a few more important insights into the obtained results to understand and appreciate them better. Specifically, we discuss the feature distribution across different grayordinates that further corroborates the importance of the proposed grayordinate ensemble strategy, followed by the subject-wise prediction analysis across different tasks. Furthermore, the predicted vs. actual activation map visualization is presented for qualitative visual inspection of the prediction performance. Finally, the generalization capabilities and robustness of the model are evaluated using cross-dataset generalization and noise error analysis, concluding with the computational analysis.

#### 4.1.1 Feature distribution

**Figure 7:**
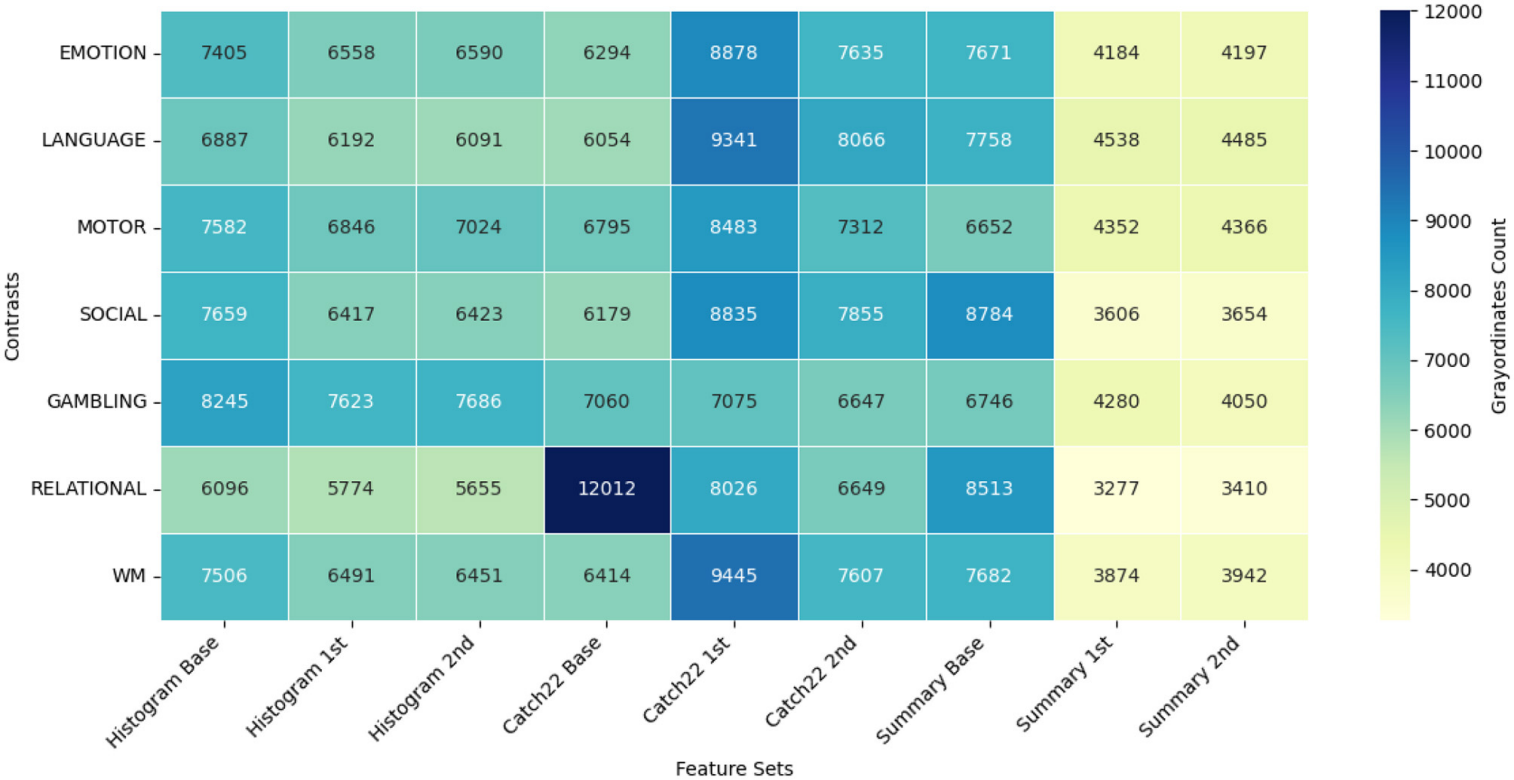
Grayordiante selection frequency heatmap across temporal feature sets and task contrasts.

**Figure 8:**
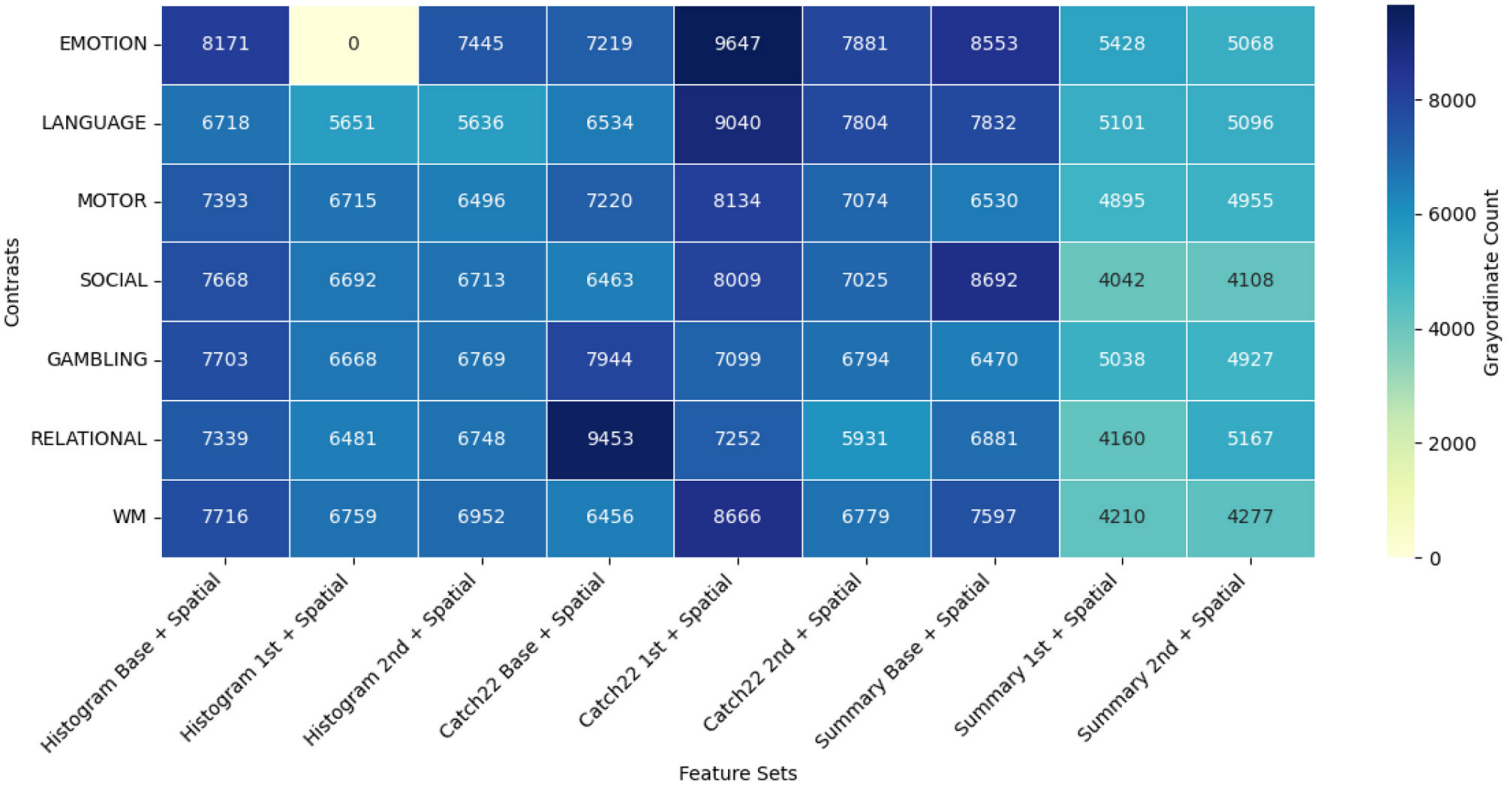
Grayordiante selection frequency heatmap across spatio-temporal feature sets and task contrasts.

To understand and explain the advantage of exploiting the different feature sets through the proposed GEMReg framework, here we studied feature distribution for grayordinate-wise prediction across seven contrasts using a diverse set of temporal and spatio-temporal features. The temporal feature sets included Histogram-based, CATCH22, and Summary statistics, each further divided into base, first-order, and second-order variants. In parallel, their spatio-temporal counterparts were also included in the analysis. As explained earlier in 3.3, for each of the 59,412 cortical grayordinates, the best-performing feature set was selected based on MSE. Figures 7, 8 depict the resulting frequency heatmap of feature distribution for temporal and spatio-temporal features, respectively.

These heatmaps reveal that different feature sets contribute variably across contrasts and brain regions, with no single representation dominating across all. Notably, the proposed histogram-based features achieved superior correlation performance in over 20, 000 grayordinates, reasserting their utility in the present prediction application. The overall findings reinforce the importance of adopting a tailored, grayordinate-specific feature selection strategy rather than applying a single feature set uniformly across all grayordinates.

#### 4.1.2 Subject-wise prediction performance

**Figure 9:**
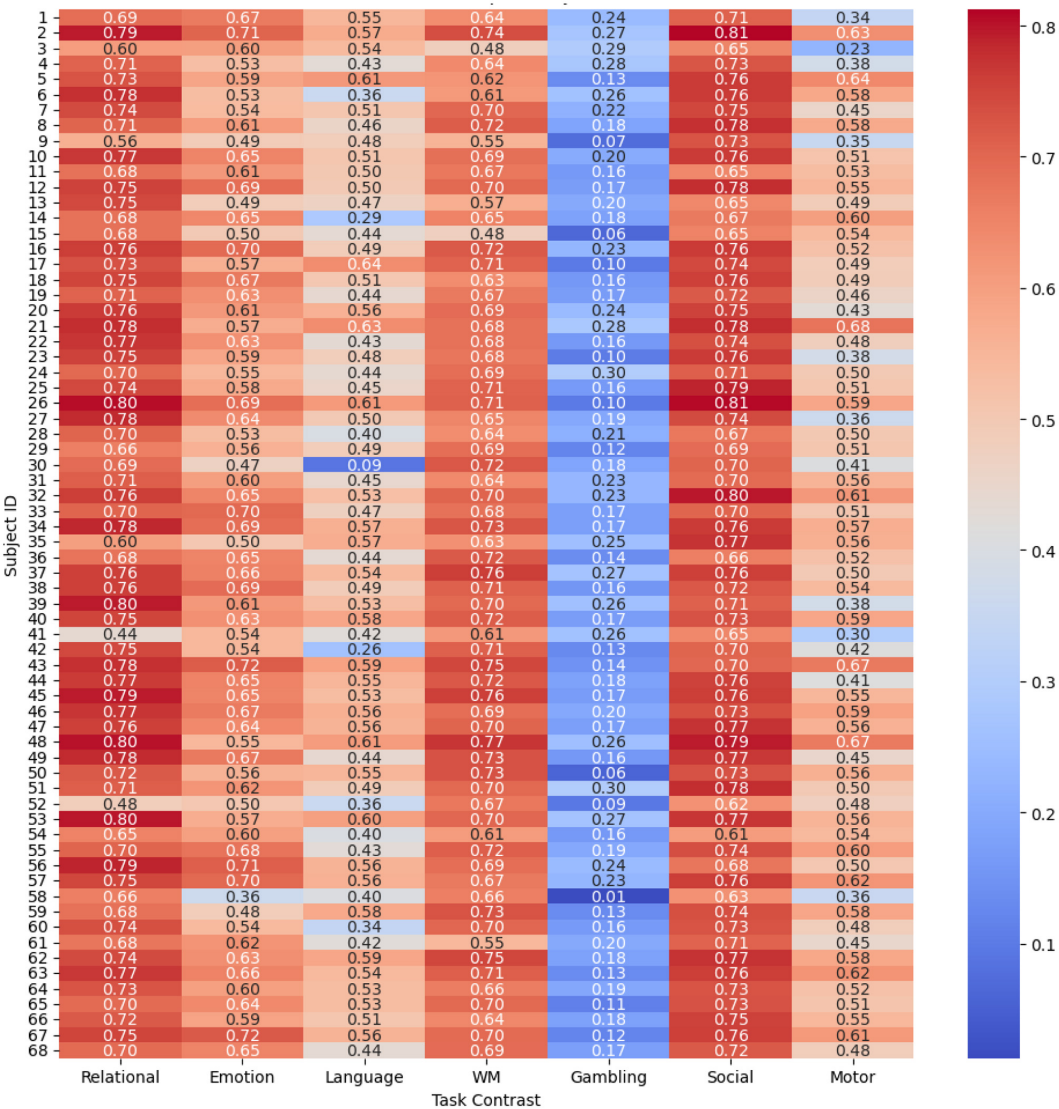
Correlations across different subjects and tasks using spatio-temporal GEMReg.

**Figure 10:**
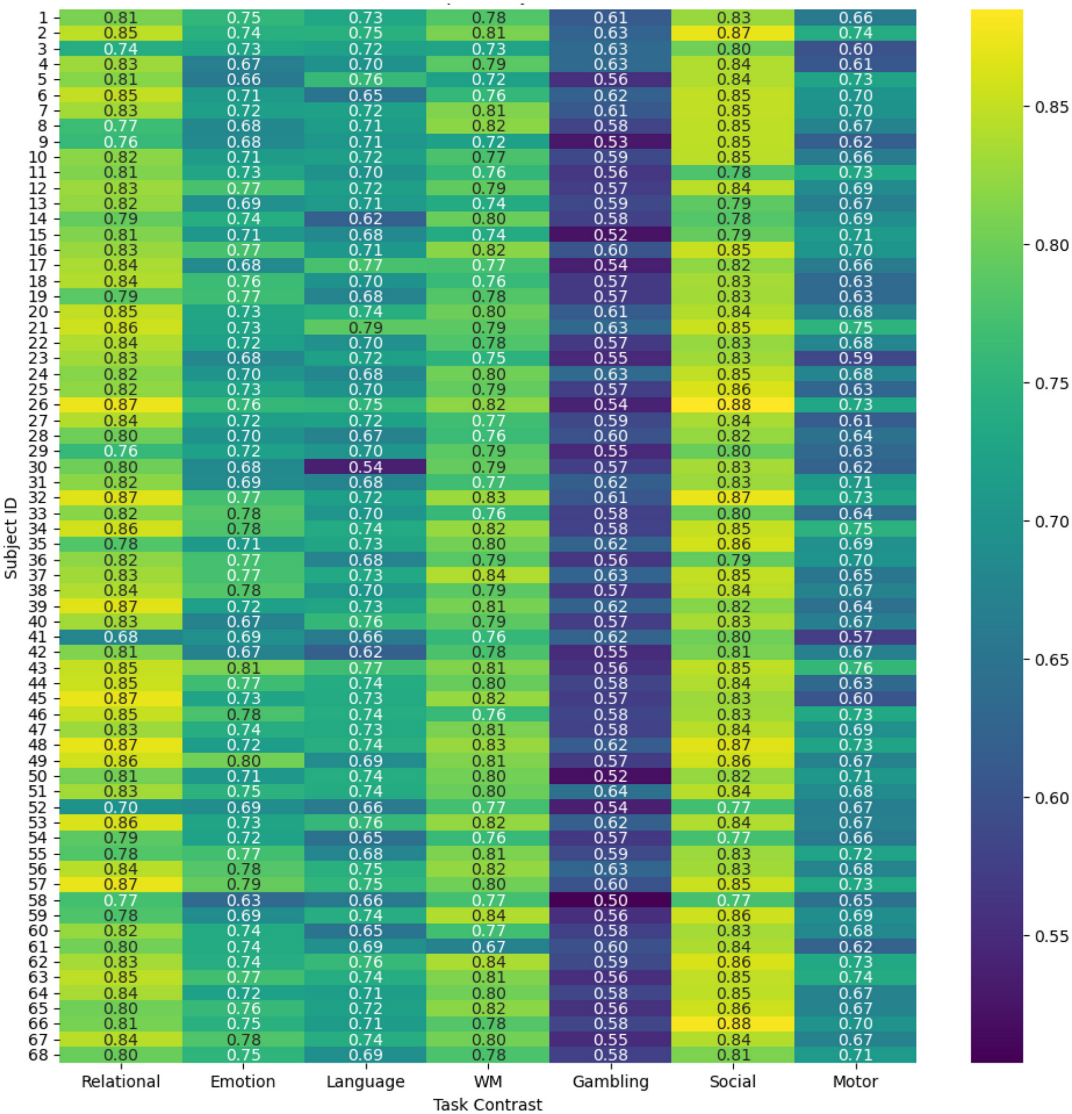
AUC scores across different subjects and tasks using spatio-temporal GEMReg.

**Figure 11:**
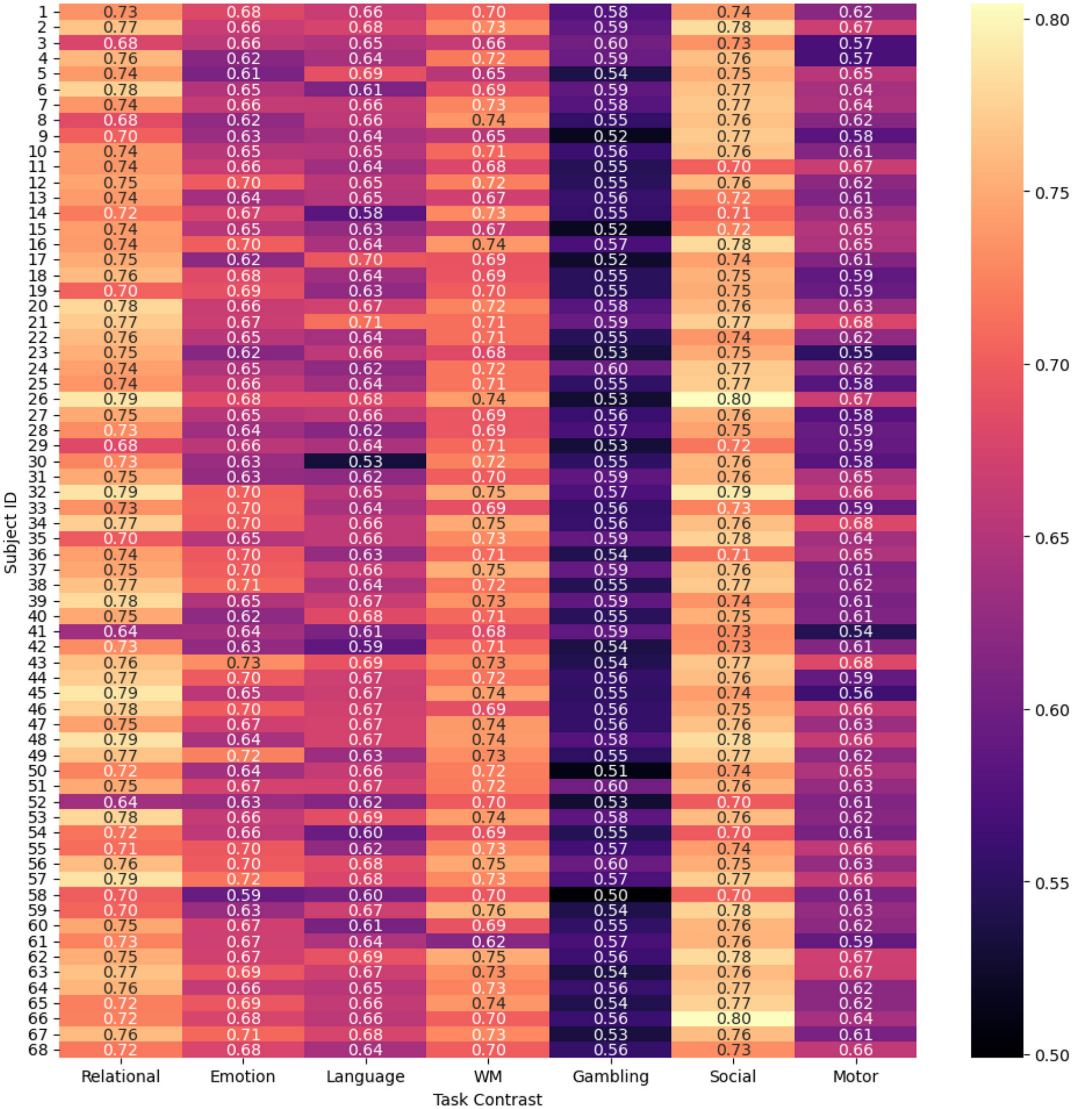
Dice coefficients across different subjects and tasks using spatio-temporal GEMReg.

To evaluate and compare the accuracy of the proposed spatio-temporal GEMReg across individual subjects, it's a subject-wise performance analyses using different metrics are provided in Figures 9–11. In Particular, Figure 9 presents a heatmap of Pearson correlation coefficients between predicted and actual activation maps across 68 subjects and seven task contrasts. The results reveal consistently good performance across subjects for RELATIONAL, SOCIAL, and WORKING MEMORY tasks, with many individuals showing correlations above 0.7. In contrast, the GAMBLING task exhibits lower correlations, suggesting greater inter-subject variability or task complexity. Similarly, Figure 10 presents the AUC scores across 68 subjects and seven task contrasts, illustrating the classification performance at an individual level. The model consistently achieves high AUC values above 0.80 across the RELATIONAL, SOCIAL, and WORKING MEMORY tasks, indicating strong discriminative ability for most subjects. In contrast, the GAMBLING task generally yields lower AUC scores, reflecting greater task complexity or variability. Finally, Figure 11 displays the Dice coefficients for each of the 68 subjects across the seven task contrasts, highlighting the overlap between predicted and actual activation regions. Again, the model achieves consistently high Dice scores, typically above 0.70, for *RELATIONAL, SOCIAL*, and *WORKING MEMORY* tasks, indicating strong spatial agreement. As seen in prior metrics, the *GAMBLING* task remains more challenging, with comparatively lower overlap scores. Overall, the analysis highlights the model's robust generalization across subjects, while also reflecting contrast-specific differences in prediction accuracy.

#### 4.1.3 Visualization and qualitative assessment

**Figure 12:**
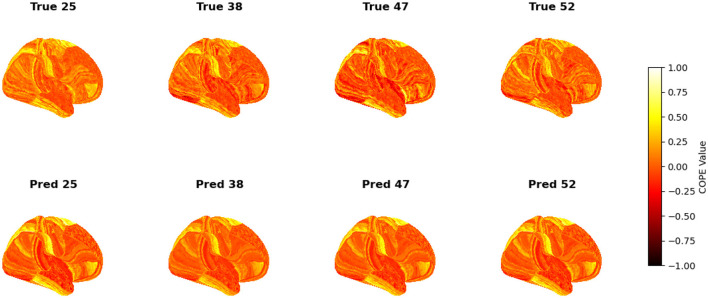
Actual vs. predicted task activation maps for RELATIONAL task contrast.

**Figure 13:**
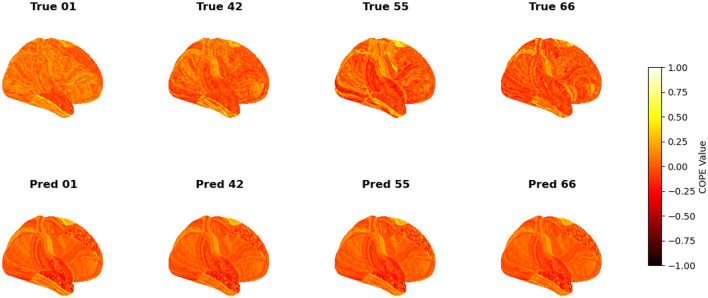
Actual vs. predicted task activation maps for EMOTION task contrast.

**Figure 14:**
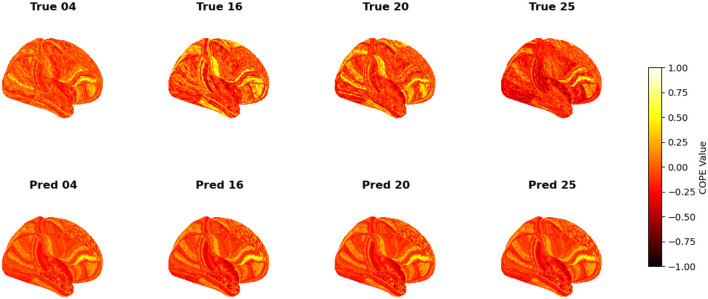
Actual vs. predicted task activation maps for LANGUAGE task contrast.

**Figure 15:**
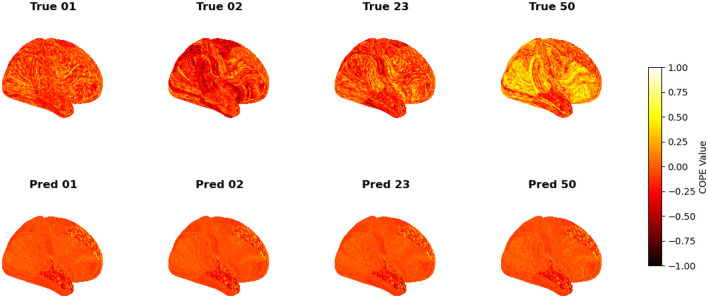
Actual vs. predicted task activation maps for GAMBLING task contrast.

**Figure 16:**
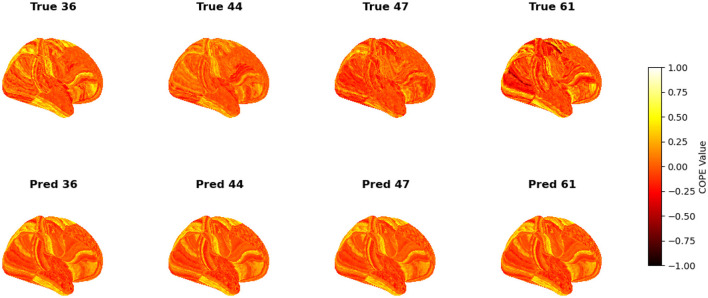
Actual vs. predicted task activation maps for WORKING MEMORY task contrast.

**Figure 17:**
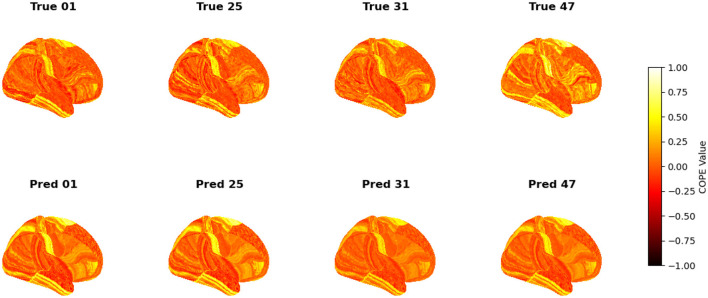
Actual vs. predicted task activation maps for SOCIAL task contrast.

**Figure 18:**
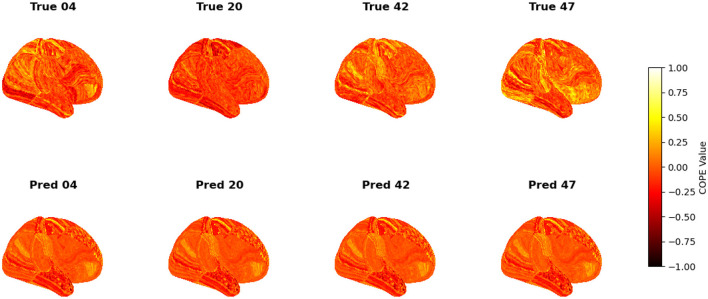
Actual vs. predicted task activation maps for MOTOR task contrast.

To visualize the subject-level performance of predicted brain activations, a surface-based visualization pipeline was implemented that compares actual and predicted activation maps projected onto the cortical surface. Such subject-specific visual comparisons are critical for identifying localized regions of strength or weakness in prediction and serve as an important complement to quantitative evaluation metrics such as Pearson correlation, MAE, and MSE. Moreover, the visual correspondence between predicted and true maps offers insights into lateralization effects and the degree to which the model captures meaningful functional topography. Using the nilearn.plotting.plot_surf_stat_map function, statistical activation values were mapped onto the inflated cortical surfaces for the right hemisphere, although a similar visualization can be obtained for the left hemisphere. The cortical geometry was defined using fs_LR 32k resolution surface mesh files for each hemisphere. For each subject, true and predicted activation maps were plotted one below the other, allowing a direct visual assessment of spatial similarity. Figures 12–18 show the representative results for a few subjects for RELATIONAL, EMOTION, LANGUAGE, GAMBLING, WORKING MEMORY, SOCIAL, and MOTOR contrasts, respectively. The strong spatial correspondence observed between the predicted and actual maps across subjects in Figure 12 demonstrates the model's ability to accurately capture subject-specific functional activation patterns in the context of the RELATIONAL task. Although EMOTION contrast results in Figure 13 reveal relatively greater discrepancies between predicted and actual activations compared to the RELATIONAL contrasts, the predicted maps still capture essential spatial features of the underlying activation patterns. Notably, common activation regions are preserved in the predictions, albeit with slightly reduced intensity, indicating that the model maintains spatial specificity despite the increased variability and complexity inherent to emotional processing. The predicted activation maps for the LANGUAGE task shown in Figure 14 exhibit substantial overlap with the ground truth, particularly within language-related cortical regions. Although slight discrepancies in activation intensity are present, the model effectively preserves the spatial organization and hemispheric lateralization typically associated with language processing. Compared to other task contrasts, the predicted activation maps for the GAMBLING task, depicted in Figure 15, exhibit relatively lower intensity and reduced spatial variability, indicating a more conservative model response in capturing task-specific patterns. Nevertheless, the predicted maps still preserve the broader spatial organization of the actual activations, particularly within ventromedial and orbitofrontal regions commonly associated with reward processing. For the working memory task, the predicted activation maps included in Figure 16 show strong spatial concordance with the ground truth, particularly in the dorsolateral prefrontal and parietal regions–areas characteristically involved in working memory processes. This consistency across subjects underscores the model's ability to reliably reconstruct activation patterns associated with higher-order executive functions, despite the distributed and complex architecture of the WORKING MEMORY network. Similarly, the predicted activation maps for the Social task in Figure 17 demonstrate a high degree of spatial correspondence with the ground truth, particularly in temporoparietal and medial prefrontal regions commonly associated with social cognition. The model effectively captures the distributed activation patterns elicited by the SOCIAL task, reinforcing its ability to generalize across subjects and task-specific cognitive domains. Finally, the prediction results in Figure 18 for the MOTOR task reveal the strong spatial agreement with the ground truth, particularly within primary motor and somatosensory regions. These results highlight the model's capacity to capture robust and localized patterns of motor-related brain activity, consistent with the known functional architecture of the MOTOR task. The overall findings presented above further demonstrate the proposed GEMReg's ability to generalize across diverse cognitive tasks, though with varying levels of predictive fidelity influenced by task complexity and inter-subject variability.

After thoroughly evaluating the prediction performance of the proposed GEMReg on the HCP dataset, we next extended its evaluation to different datasets and varying signal qualities to verify its generalizability and robustness to out-of-sample and non-compliant subjects. For the non-compliant subjects, since the required fMRI data are not publicly available, we conducted performance analysis using signals with varying degrees of head motion, which serves as a natural proxy for noisy signal acquisition arising in such subjects.

#### 4.1.4 Cross-dataset generalization: evaluation on the CHCP dataset

To further assess the robustness and generalizability of the proposed GEMReg model, we conducted an independent evaluation using the Chinese Human Connectome Project (CHCP) (Yang et al., 2024) dataset, which differs substantially from the Human Connectome Project (HCP) dataset used for training. Specifically, GEMReg was tested on 40 independent CHCP subjects, each with resting-state fMRI (rs-fMRI) data comprising 634 time points. In contrast, the HCP dataset includes 1,200 time points per subject. Furthermore, the CHCP dataset employed distinct scanner hardware, acquisition parameters, and preprocessing pipelines, providing a rigorous evaluation of the model's ability to generalize across datasets with varying acquisition characteristics and temporal resolutions. The quantitative performance metrics of the GEMReg on the CHCP dataset are summarized in Table 4. Comparing these results with the results on the HCP dataset (tabulated in Table 3), clearly indicates that the GEMReg model, trained on the HCP dataset, maintains strong predictive performance on the CHCP dataset as well, yielding correlations that are comparable to or slightly higher than those obtained on HCP test subjects for several task contrasts, and only marginally lower for others. Specifically, GEMReg achieved superior predictive accuracy for the MOTOR and EMOTION tasks, while performance on the GAMBLING task was considerably reduced, likely due to the high degree of inter-subject variability inherent in this task paradigm. These findings underscore a key advantage of the GEMReg framework—its capacity to extract intrinsic temporal features from rs-fMRI time series rather than relying on a fixed number of time samples that may change with the sampling frequency during signal acquisition. Overall, this cross-dataset evaluation demonstrates that GEMReg is robust to differences in scanner protocols and temporal resolution, and capable of generalizing its learned representations to entirely independent datasets without retraining. This makes it a scalable and transferable framework for predicting task-related brain activations from resting-state fMRI across diverse populations and imaging protocols.

**Table 4:** Cross-generalization on CHCP dataset using spatio-temporal GEMReg model.

**Contrasts**	**r**	**DICE**	**AUC**
RELATIONAL	0.7072	0.7015	0.7813
EMOTION	0.6487	0.6890	0.7553
LANGUAGE	0.4618	0.6009	0.6912
GAMBLING	0.0711	0.4837	0.5053
SOCIAL	0.6878	0.6929	0.7601
MOTOR	0.5388	0.6212	0.6801

#### 4.1.5 Effect of signal quality on prediction performance

**Figure 19:**
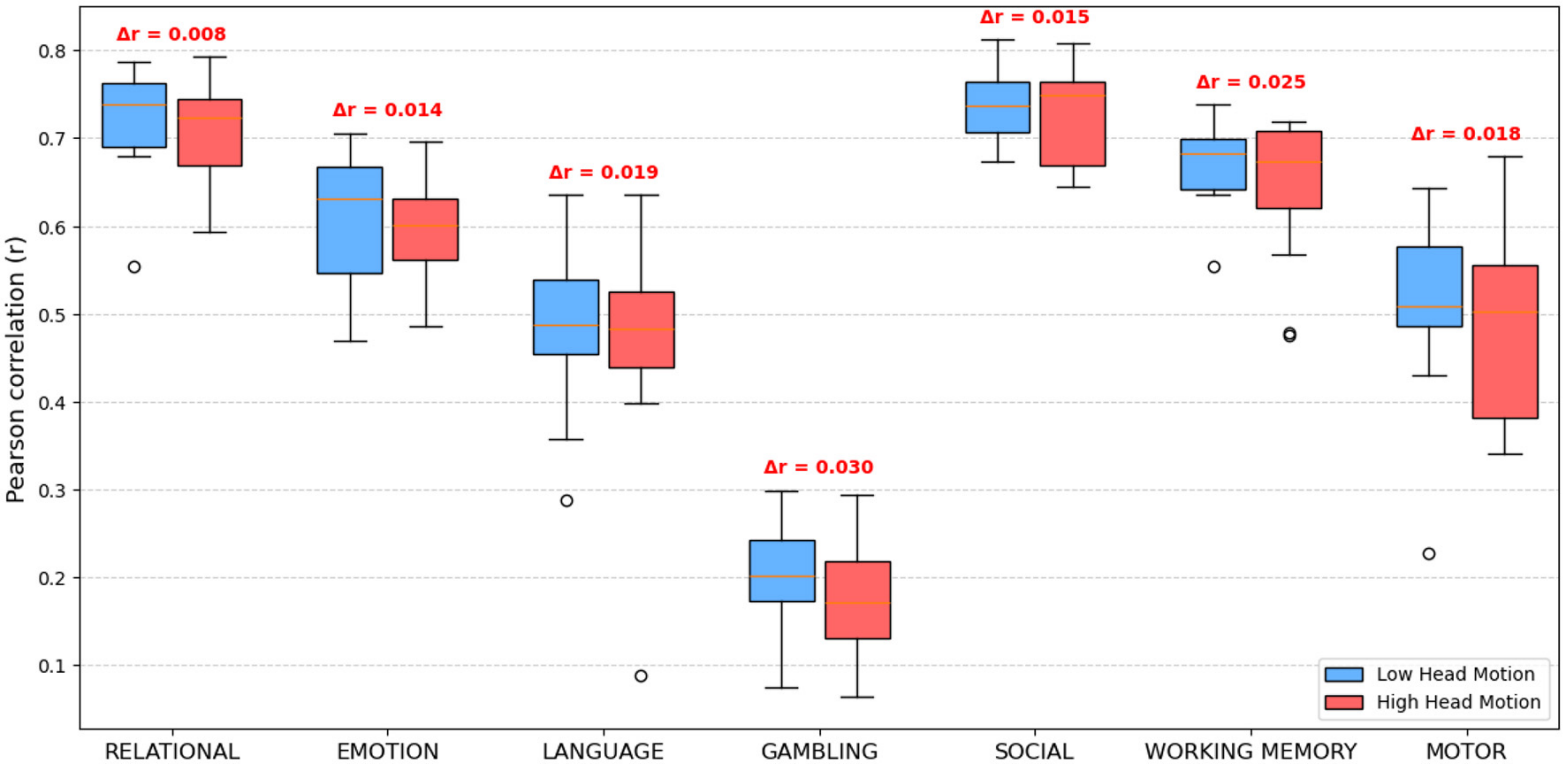
Boxplots of predictive performance (Pearson *r*) for low and high head motion subjects across seven task contrasts. Δ*r* represents the mean correlation difference between groups.

To assess the robustness of the proposed spatio-temporal GEMReg framework to varying signal quality, predominantly arising from the motion-related noise, we investigated the relationship between head motion during resting-state fMRI acquisition and the accuracy of predicted task activation maps. Although our dataset comprised healthy young adults from the HCP, head motion was used as a natural proxy for noise that would typically arise in populations for whom task-fMRI acquisition is challenging, such as elderly or clinical groups. Importantly, no artificial noise was introduced; instead, we quantified naturally occurring head motion using the mean framewise displacement (FD) derived from each participant's motion regressors. Mean FD was computed following the approach of Power et al. (2012), as the sum of absolute frame-to-frame differences across six motion parameters. Subjects were divided into two equal-sized groups, Low Head Motion and High Head Motion, based on the median FD (i.e., the 50th percentile). The predictive performance of GEMReg was then compared between the two groups across all seven task contrasts. For each subject, the Pearson correlation coefficient (*r*) between the predicted and actual task activation maps was computed, and group-wise performance was summarized using boxplots as shown in Figure 19. Across all task contrasts, the model exhibited a small but consistent reduction in prediction accuracy for subjects with higher head motion, with a mean drop in correlation of 0.018. Furthermore, similar analysis was extended to other prediction models, viz., the proposed histogram-based model, temporal GEMReg, and Tavor et al. (2016). All models exhibited a similar decline in prediction accuracy, with mean drops of 0.0185, 0.018, and 0.016, respectively. Overall, these findings demonstrate that while head motion has a measurable effect on predictive accuracy, the spatio-temporal GEMReg framework still performs the best and hence could be extended to more challenging cohorts, provided that appropriate motion correction and artifact mitigation procedures are applied during preprocessing.

#### 4.1.6 Computational analysis

All experiments presented above were performed on a system with an Intel Xeon Platinum 8352Y CPU (2.20 GHz, 2 processors), 256 GB of RAM (3,200 MHz), and an NVIDIA RTX A6000 GPU (47 GB of VRAM). Table 5 summarizes the average training and inference times required for the histogram-based, CATCH22-based, and Summary-based models, along with the overall GEMReg framework. The training time corresponds to the duration required for grayordinate-wise model fitting across all 59,412 grayordinates, whereas the inference time represents the average duration required to generate activation predictions for a test subject. The results demonstrate that, while the histogram-based component required the longest training duration due to its higher feature dimensionality, the inference time remained consistently short (within 2 minutes) across all models. This highlights the efficiency and scalability of GEMReg, making it well-suited for large-scale or cross-dataset neuroimaging analyses where computational cost is a critical factor.

**Table 5:** Training and inference times for individual feature-based components and the overall GEMReg framework.

**S. No**.	**Model**	**Training time**	**Inference time**
1	Histogram-based model	1 h	14 s
2	CATCH22-based model	45 min	10 s
3	Summary-based model	30 min	9 s
4	GEMReg	9 h	2 min

Finally, in the following section, we present an ablation study to dissect the individual contributions of each component in our modeling pipeline and provide deeper insights into how various feature-regressor choices impact overall prediction performance.

### 4.2 Ablation study

#### 4.2.1 Selection of optimal CNN for histogram-based features

As mentioned earlier in Section 4.1, three different 2D-CNN architectures were designed for regression using the proposed histogram features to find the optimal one. CNN1, our baseline model, consisted of two convolutional layers with 16 and 32 filters of size 3 × 3, respectively, the first layer including padding of 1, each followed by ReLU activation. The feature maps were then reduced using an adaptive average pooling layer to a fixed size of 5 × 10 per channel, flattened into a vector, and passed through two fully connected layers, with 128 hidden units in the first layer and a single scalar output in the second layer representing the predicted activation value. CNN2 employed two convolutional layers with 32 and 64 filters, a larger first filter of 5 × 5 with padding of 2, followed by ReLU activation, and applied max pooling to downsample the feature maps. The flattened vector was then passed through two fully connected layers with 256 hidden units in the first layer and a single scalar output. CNN3 introduced a third convolutional layer, using 16, 16, and 32 filters for the successive layers, followed by ReLU activation, and adaptive average pooling reduces the feature maps to 4 × 8 per channel. Similar to CNN1, the flattened features were passed through two fully connected layers with 128 hidden units in the first layer and a single scalar output. Among these architectures, CNN1 consistently outperformed CNN2 and CNN3 when trained on the histogram-based feature representations, indicating that a relatively shallow architecture with appropriately sized filters and adaptive pooling is sufficient to capture the temporal characteristics of the input features while avoiding overfitting. The correlation results of histogram-based features are provided in Tables 6, 7.

**Table 6:** Correlations using the proposed histogram-based features with different CNN architectures.

**Contrast**	**Features**	**r of CNN1**	**r of CNN2**	**r of CNN3**
RELATIONAL	HIST	0.7100	0.7072	0.7054
	1st HIST	0.7111	0.7095	0.7062
	2nd HIST	0.7120	0.7095	0.7037
EMOTION	HIST	0.5901	0.5882	0.5861
	1st HIST	0.5899	0.5873	0.5835
	2nd HIST	0.5932	0.5910	0.5818
LANGUAGE	HIST	0.4701	0.4638	0.4676
	1st HIST	0.4699	0.4658	0.4655
	2nd HIST	0.4721	0.4703	0.4671
GAMBLING	HIST	0.1109	0.1081	0.1063
	1st HIST	0.0899	0.0880	0.0860
	2nd HIST	0.1120	0.1101	0.1070
SOCIAL	HIST	0.7210	0.7188	0.7165
	1st HIST	0.7209	0.7182	0.7157
	2nd HIST	0.7211	0.7181	0.7166
WM	HIST	0.6662	0.6638	0.6601
	1st HIST	0.6601	0.6578	0.6551
	2nd HIST	0.6671	0.6642	0.6619
MOTOR	HIST	0.4998	0.4978	0.4942
	1st HIST	0.4903	0.4881	0.4858
	2nd HIST	0.5020	0.4993	0.4968

**Table 7:** Correlations using the combined histogram and spatial connectivity features with different CNN architectures.

**Contrast**	**Features**	**r of CNN1**	**r of CNN2**	**r of CNN3**
RELATIONAL	H + S	0.7106	0.7078	0.7059
	1st H + S	0.7117	0.7101	0.7067
	2nd H + S	0.7125	0.7101	0.7043
EMOTION	H + S	0.5906	0.5888	0.5866
	1st H + S	0.5904	0.5879	0.5840
	2nd H + S	0.5938	0.5916	0.5823
LANGUAGE	H + S	0.4706	0.4643	0.4681
	1st H + S	0.4705	0.4663	0.4660
	2nd H + S	0.4726	0.4709	0.4676
GAMBLING	H + S	0.1115	0.1087	0.1068
	1st H + S	0.0904	0.0886	0.0865
	2nd H + S	0.1126	0.1107	0.1075
SOCIAL	H + S	0.7216	0.7193	0.7171
	1st H + S	0.7215	0.7187	0.7163
	2nd H + S	0.7217	0.7187	0.7172
WM	H + S	0.6667	0.6643	0.6606
	1st H + S	0.6606	0.6583	0.6556
	2nd H + S	0.6677	0.6648	0.6624
MOTOR	H + S	0.5003	0.4983	0.4947
	1st H + S	0.4908	0.4886	0.4863
	2nd H + S	0.5026	0.4999	0.4973

H + S refers to histogram + spatial concatenated features.

#### 4.2.2 Selection of optimal temporal feature extractors

As described in Section 2.3, we evaluated several state-of-the-art time series feature extraction methods to identify effective temporal descriptors for predicting task activation maps from resting-state fMRI data. The assessed methods included MrHydra, DRCIF, HYDRA, ROCKET, RISE, CATCH22, and the SUMMARY feature set.

The comparative results in Table 8 show that CATCH22 and SUMMARY features achieved the best predictive performance, yielding higher correlations across task contrasts. Consequently, these two feature sets were selected for subsequent modeling of task activation maps.

**Table 8:** Prediction of task activation maps using different time series feature extractors (average correlation across subjects).

**Features**	**RELATIONAL**	**EMOTION**	**LANGUAGE**	**GAMBLING**	**SOCIAL**	**WM**	**MOTOR**
MRHYDRA	0.6921	0.5647	0.4402	0.0783	0.6981	0.6425	0.4587
DRCIF	0.6805	0.5594	0.4378	0.0724	0.6902	0.6371	0.4520
ROCKET	0.6901	0.5623	0.4427	0.0741	0.6955	0.6413	0.4575
HYDRA	0.6887	0.5581	0.4384	0.0702	0.6920	0.6364	0.4508
RISE	0.6911	0.5601	0.4395	0.0715	0.6938	0.6392	0.4541
CATCH22	**0.7110**	**0.5823**	**0.4530**	**0.0883**	**0.7134**	**0.6582**	**0.4759**
SUMMARY	**0.7110**	**0.5924**	**0.4691**	**0.1107**	**0.7203**	**0.6651**	**0.4905**

The bold rows indicate the best performing features.

#### 4.2.3 Selection of optimal feature-regressor combinations

As discussed earlier in Section 3.3, each of the proposed activation map prediction models responded differently when trained on distinct feature sets, highlighting the variability in predictive capacity depending on the type of information used. To better understand these differences and identify the optimal modeling strategy, a detailed ablation study was conducted as presented below. Starting with the temporal features, Table 9 summarizes the correlation results obtained across various task contrasts when nine distinct feature sets: HISTOGRAM, 1*st* order HISTOGRAM, 2*nd* order HISTOGRAM, CATCH22, 1*st* order CATCH22, 2*nd* order CATCH22, SUMMARY, 1*st* order SUMMARY, and 2nd order SUMMARY, were applied to different regressors–LASSO, LR, SVR, Ridge, XGBoost, Gradient Boosting and CNN1. Overall, across all task contrasts and the existing temporal feature sets, SVR consistently achieved the highest correlation values, demonstrating its robustness and generalizability in modeling brain activation patterns from temporal features. Among the remaining models, LASSO showed competitive performance–especially with CATCH22-based features–occasionally approaching or matching SVR's performance. For the proposed histogram-based features, CNN1 outperformed in all three representations. Furthermore, to obtain the optimal prediction for each grayordinate, the best-performing feature-regressor combinations, demonstrating the highest average Pearson correlation coefficients, were identified from Tables 6, 9. These included the raw histogram, first-order histogram, and second-order histogram feature sets (all using CNN1); the raw-CATCH22 set (SVR); the first- and second-order CATCH22 sets (both using LASSO); and the raw, first-order, and second-order SUMMARY sets (all using SVR). Subsequently, as detailed in Section 3.3, a grayordinate ensemble strategy (GEMReg) was implemented, wherein predictions from all the nine combinations were evaluated for each grayordinate using four performance metrics—*r*, *r*^2^, MAE, and MSE. The combination achieving the best metric was selected as the optimal predictor for that grayordinate. Table 10 summarizes the results of this optimal selection strategy using different metrics across various cognitive task contrasts. The results indicate that the MSE-based optimal selection approach performs the best, consistently outperforming the others across multiple metrics and tasks. The superior performance of this optimal selection approach over the individual feature-based models also validated the benefit of optimal temporal feature selection tailored to localized brain dynamics.

**Table 9:** Correlations for temporal feature sets across task contrasts using different regressors.

**Contrast**	**Features**	**LASSO**	**LR**	**SVR**	**RIDGE**	**XGB**	**GB**
RELATIONAL	CATCH22	0.707	0.700	0.711	0.630	0.670	0.640
	1st order CATCH22	0.710	0.690	0.705	0.650	0.650	0.660
	2nd order CATCH22	0.710	0.690	0.705	0.630	0.670	0.640
	SUMMARY	0.711	0.705	0.711	0.650	0.650	0.660
	1st order SUMMARY	0.710	0.704	0.710	0.630	0.670	0.640
	2nd order SUMMARY	0.710	0.703	0.711	0.650	0.650	0.660
EMOTION	CATCH22	0.579	0.483	0.5823	0.422	0.469	0.493
	1st order CATCH22	0.590	0.485	0.5880	0.425	0.470	0.494
	2nd order CATCH22	0.5888	0.487	0.5870	0.420	0.462	0.500
	SUMMARY	0.591	0.492	0.5924	0.440	0.464	0.480
	1st order SUMMARY	0.589	0.489	0.5904	0.420	0.474	0.500
	2nd order SUMMARY	0.590	0.490	0.5905	0.440	0.462	0.500
LANGUAGE	CATCH22	0.4526	0.369	0.4530	0.346	0.361	0.357
	1st order CATCH22	0.4659	0.379	0.4630	0.326	0.381	0.337
	2nd order CATCH22	0.4646	0.378	0.4630	0.346	0.361	0.357
	SUMMARY	0.4687	0.386	0.4691	0.326	0.381	0.337
	1st order SUMMARY	0.4688	0.384	0.4673	0.346	0.361	0.357
	2nd order SUMMARY	0.4682	0.385	0.4674	0.336	0.371	0.347
GAMBLING	CATCH22	0.0820	0.0643	0.0883	0.0791	0.0754	0.0731
	1st order CATCH22	0.1020	0.0650	0.1010	0.0801	0.0744	0.0741
	2nd order CATCH22	0.1009	0.0649	0.1008	0.0781	0.0764	0.0721
	SUMMARY	0.1106	0.0520	0.1107	0.0791	0.0754	0.0731
	1st order SUMMARY	0.1075	0.0501	0.1076	0.0781	0.0764	0.0721
	2nd order SUMMARY	0.1073	0.0512	0.1074	0.0801	0.0744	0.0741
SOCIAL	CATCH22	0.7130	0.6200	0.7134	0.6421	0.6371	0.6120
	1st order CATCH22	0.7172	0.6319	0.7168	0.6431	0.6361	0.6130
	2nd order CATCH22	0.7166	0.0649	0.7160	0.6411	0.6381	0.6110
	SUMMARY	0.7200	0.6500	0.7203	0.6421	0.6371	0.6120
	1st order SUMMARY	0.7172	0.6498	0.7180	0.6431	0.6361	0.6130
	2nd order SUMMARY	0.7177	0.6497	0.7180	0.6411	0.6381	0.6110
WM	CATCH22	0.657	0.512	0.6582	0.577	0.582	0.583
	1st order CATCH22	0.6631	0.592	0.6610	0.597	0.562	0.603
	2nd order CATCH22	0.6623	0.690	0.6610	0.587	0.572	0.593
	SUMMARY	0.663	0.520	0.6651	0.577	0.582	0.583
	1st order SUMMARY	0.662	0.501	0.6646	0.597	0.562	0.603
	2nd order SUMMARY	0.662	0.512	0.6647	0.587	0.572	0.593
MOTOR	CATCH22	0.4757	0.3521	0.4759	0.4031	0.4126	0.4112
	1st order CATCH22	0.4867	0.3519	0.4861	0.4041	0.4116	0.4122
	2nd order CATCH22	0.4855	0.3517	0.4852	0.4021	0.4136	0.4102
	SUMMARY	0.4900	0.3519	0.4905	0.4031	0.4126	0.4112
	1st order SUMMARY	0.4865	0.3610	0.4866	0.4041	0.4116	0.4122
	2nd order SUMMARY	0.4863	0.3600	0.4867	0.4021	0.4136	0.4102

**Table 10:** Performance metrics for optimally selected temporal features using different accuracy measures.

**Task**	**Metric**	***r***	**DICE**	**AUC**
RELATIONAL	r	0.7101	0.7215	0.8013
	*r* ^2^	0.7050	0.7170	0.7980
	MAE	0.7100	0.7195	0.8090
	MSE	0.7201	0.7317	0.8101
EMOTION	r	0.5940	0.6500	0.7200
	*r* ^2^	0.5750	0.6400	0.7050
	MAE	0.6000	0.6530	0.7190
	MSE	0.6042	0.6590	0.7253
LANGUAGE	r	0.4600	0.6200	0.6900
	*r* ^2^	0.4400	0.6100	0.6750
	MAE	0.4550	0.6250	0.6950
	MSE	0.4746	0.6309	0.7001
GAMBLING	r	0.1200	0.5200	0.5500
	*r* ^2^	0.0700	0.5100	0.5300
	MAE	0.1100	0.5300	0.5400
	MSE	0.1430	0.5387	0.5553
SOCIAL	r	0.7100	0.7300	0.8200
	*r* ^2^	0.7000	0.7200	0.8100
	MAE	0.7150	0.7280	0.8205
	MSE	0.7258	0.7329	0.8091
WM	r	0.6400	0.7000	0.7600
	*r* ^2^	0.6300	0.6900	0.7500
	MAE	0.6400	0.6950	0.7600
	MSE	0.6560	0.7011	0.7660
MOTOR	r	0.4800	0.6000	0.6600
	*r* ^2^	0.4600	0.5900	0.6400
	MAE	0.4750	0.5980	0.6500
	MSE	0.5029	0.6012	0.6601

Finally, an ablation study was conducted to evaluate the performance of various regression models employed earlier using different combinations of temporal and spatial features across all contrasts. The features analyzed included histogram-based features (HIST), CATCH22 time series features, and SUMMARY statistical descriptors, each combined with spatial features. First and second derivatives of these temporal features were also incorporated to assess their incremental value. The results obtained for these different feature-regressor combinations are tabulated in Tables 7, 11. Following this, the same optimal selection strategy, as employed for the temporal features, was applied for these spatio-temporal feature sets. Performance metrics resulting from this procedure are presented in Table 12, which summarizes the optimal regression results across various cognitive task contrasts. These results demonstrate that the ensemble-based optimal feature selection approach using MSE again performs the best, providing significant improvements over individual feature sets. Furthermore, compared to spatial-only or temporal-only features, the use of spatio-temporal features enhances the predictive modeling of task-related brain activity, highlighting the value of combining spatial structure with temporal dynamics at the level of individual grayordinates.

**Table 11:** Correlations for spatio-temporal feature sets across task contrasts using different regressors.

**Contrast**	**Features**	**LASSO**	**LR**	**SVR**	**RIDGE**	**XGB**	**GB**
RELATIONAL	CATCH22 + SPATIAL	0.711	0.500	0.705	0.682	0.621	0.692
	1st CATCH22 + SPATIAL	0.710	0.490	0.705	0.642	0.645	0.657
	2nd CATCH22 + SPATIAL	0.709	0.500	0.705	0.637	0.666	0.667
	SUMMARY + SPATIAL	0.711	0.520	0.712	0.667	0.651	0.687
	1st SUMMARY + SPATIAL	0.710	0.520	0.711	0.682	0.615	0.677
	2nd SUMMARY + SPATIAL	0.709	0.520	0.711	0.657	0.636	0.647
EMOTION	CATCH22 + SPATIAL	0.5823	0.3725	0.5821	0.4997	0.4635	0.4872
	1st CATCH22 + SPATIAL	0.5908	0.3730	0.5900	0.4031	0.4782	0.4026
	2nd CATCH22 + SPATIAL	0.5888	0.3733	0.5875	0.4981	0.4929	0.4675
	SUMMARY + SPATIAL	0.5930	0.3789	0.5931	0.4085	0.4691	0.4868
	1st SUMMARY + SPATIAL	0.5903	0.3788	0.5904	0.4013	0.4955	0.4832
	2nd SUMMARY + SPATIAL	0.5905	0.3788	0.5906	0.4869	0.4082	0.4799
LANGUAGE	CATCH22 + SPATIAL	0.4530	0.3092	0.4528	0.4019	0.3843	0.3918
	1st CATCH22 + SPATIAL	0.4659	0.3223	0.4652	0.4002	0.3857	0.3914
	2nd CATCH22 + SPATIAL	0.4646	0.3220	0.4645	0.4013	0.3860	0.3925
	SUMMARY + SPATIAL	0.4701	0.3312	0.4703	0.4008	0.3845	0.3910
	1st SUMMARY + SPATIAL	0.4671	0.3296	0.4674	0.4011	0.3852	0.3922
	2nd SUMMARY + SPATIAL	0.4670	0.3310	0.4675	0.4009	0.3851	0.3931
GAMBLING	CATCH22 + SPATIAL	0.0833	0.0217	0.0829	0.0719	0.0714	0.0701
	1st CATCH22 + SPATIAL	0.1020	0.0313	0.1019	0.0726	0.0715	0.0706
	2nd CATCH22 + SPATIAL	0.1009	0.0312	0.1007	0.0725	0.0713	0.0702
	SUMMARY + SPATIAL	0.1135	0.0451	0.1141	0.0722	0.0709	0.0704
	1st SUMMARY + SPATIAL	0.1073	0.0441	0.1078	0.0720	0.0712	0.0701
	2nd SUMMARY + SPATIAL	0.1071	0.0439	0.1076	0.0724	0.0710	0.0705
SOCIAL	CATCH22 + SPATIAL	0.7134	0.5010	0.7124	0.6410	0.6362	0.6587
	1st CATCH22 + SPATIAL	0.7172	0.5232	0.7162	0.6414	0.6365	0.6589
	2nd CATCH22 + SPATIAL	0.7166	0.5226	0.7156	0.6413	0.6363	0.6588
	SUMMARY + SPATIAL	0.7201	0.5378	0.7206	0.6412	0.6364	0.6590
	1st SUMMARY + SPATIAL	0.7176	0.5371	0.7180	0.6411	0.6362	0.6589
	2nd SUMMARY + SPATIAL	0.7175	0.5370	0.7181	0.6413	0.6364	0.6588
WM	CATCH22 + SPATIAL	0.6582	0.4311	0.6581	0.5913	0.5879	0.5723
	1st CATCH22 + SPATIAL	0.6631	0.4379	0.6630	0.5924	0.5865	0.5728
	2nd CATCH22 + SPATIAL	0.6623	0.4371	0.6617	0.5920	0.5880	0.5731
	SUMMARY + SPATIAL	0.6652	0.4407	0.6655	0.5915	0.5862	0.5725
	1st SUMMARY + SPATIAL	0.6641	0.4406	0.6646	0.5930	0.5875	0.5730
	2nd SUMMARY + SPATIAL	0.6645	0.4405	0.6647	0.5922	0.5870	0.5726
MOTOR	CATCH22 + SPATIAL	0.4759	0.3090	0.4756	0.4254	0.4119	0.4099
	1st CATCH22 + SPATIAL	0.4867	0.3093	0.4866	0.4258	0.4125	0.4102
	2nd CATCH22 + SPATIAL	0.4855	0.3126	0.4853	0.4255	0.4123	0.4100
	SUMMARY + SPATIAL	0.4912	0.3213	0.4917	0.4256	0.4120	0.4098
	1st SUMMARY + SPATIAL	0.4861	0.3210	0.4867	0.4257	0.4124	0.4103
	2nd SUMMARY + SPATIAL	0.4863	0.3210	0.4868	0.4256	0.4121	0.4101

**Table 12:** Performance metrics for optimally selected spatio-temporal features using different accuracy measures.

**Task**	**Metric**	***r***	**DICE**	**AUC**
RELATIONAL	r	0.7201	0.7312	0.8085
	*r* ^2^	0.7100	0.7280	0.8054
	MAE	0.7250	0.7310	0.8095
	MSE	0.7273	0.7321	0.8109
EMOTION	r	0.6050	0.6575	0.7265
	*r* ^2^	0.5800	0.6480	0.7095
	MAE	0.6040	0.6590	0.7270
	MSE	0.6170	0.6599	0.7301
LANGUAGE	r	0.4830	0.6400	0.7050
	*r* ^2^	0.4500	0.6300	0.6870
	MAE	0.4840	0.6350	0.7060
	MSE	0.4858	0.6369	0.7101
GAMBLING	r	0.1400	0.5300	0.5600
	*r* ^2^	0.0850	0.5200	0.5400
	MAE	0.1450	0.5350	0.5620
	MSE	0.1510	0.5406	0.5648
SOCIAL	r	0.7250	0.7400	0.8090
	*r* ^2^	0.7100	0.7350	0.8050
	MAE	0.7260	0.7405	0.8095
	MSE	0.7299	0.7427	0.8101
WM	r	0.6700	0.7070	0.7820
	*r* ^2^	0.6500	0.6990	0.7200
	MAE	0.6700	0.7090	0.7830
	MSE	0.6721	0.7102	0.7841
MOTOR	r	0.5050	0.6100	0.6600
	*r* ^2^	0.4740	0.6000	0.6480
	MAE	0.5060	0.6105	0.6610
	MSE	0.5103	0.6121	0.6652

#### 4.2.4 Contribution of the proposed histogram-based features in GEMReg

**Figure 20:**
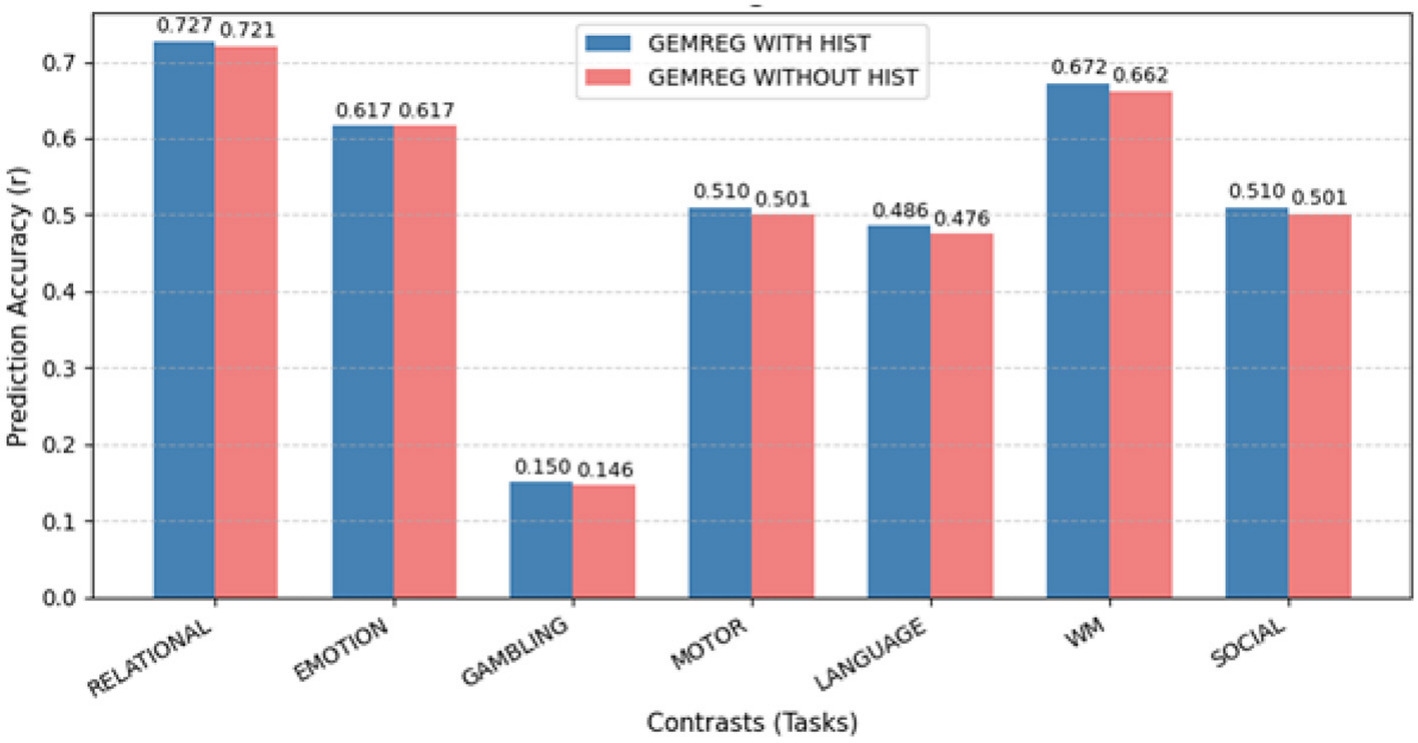
Contribution of the proposed histogram-based features in GEMReg.

To assess the contribution of the proposed histogram-based time-series features to the overall GEMReg performance, we conducted an ablation study by comparing model performance with and without these features. As shown in Figure 20, the removal of the histogram-based features led to a consistent reduction in prediction accuracy across all task contrasts. The most pronounced effect was observed for the MOTOR, LANGUAGE, SOCIAL, and WORKING MEMORY tasks, suggesting that the histogram-based features effectively capture fine-grained temporal characteristics, particularly beneficial for these cognitive domains. This further underscores the importance of the proposed histogram-based features in improving the predictive performance of the GEMReg framework, as already suggested by the results in Figure 8.

After a detailed performance analysis and the ablation study presented in this section, the next section provides the conclusions of the presented work and a few pointers for its possible extensions.

## 5 Conclusion and future scope

This study introduces a novel framework for predicting task-fMRI activation maps solely using rs-fMRI by formulating the prediction problem as a time series regression problem–a departure from traditional connectivity-based models. By extracting optimal temporal features and training 59, 412 grayordinate-specific models, the proposed GEMReg framework captures fine-grained, grayordinate-specific dynamics. Further, its integration with spatial connectivity features results in a state-of-the-art spatio-temporal GEMReg model, which consistently outperforms existing methods across evaluation metrics. This is the first demonstration of time series regression-based task activation map prediction using rs-fMRI and offers significant promise for clinical and cognitive neuroscience applications, especially for non-compliant populations.

Looking ahead, while the univariate strategy provides detailed insights, it poses computational challenges due to the large number of individual models. To address this, future work can explore multivariate modeling approaches, which can simultaneously capture interdependencies across grayordinates or brain regions. A systematic comparison between univariate and multivariate methods can help balance accuracy and efficiency in task activation map prediction. Also, although outperforming the existing methods, the proposed model showed comparatively lower correlation values for the gambling task than other contrasts. This outcome may be attributed to the inherently higher subject-level variability in neural responses during gambling tasks, as individuals differ in risk-taking tendencies, reward sensitivity, and decision-making strategies. Existing literature also reports relatively weaker prediction performance and lower consistency for the gambling contrast, supporting our observation. Future work could therefore focus on developing models that explicitly account for such inter-individual variability, potentially incorporating subject-specific behavioral or personality measures, to improve the robustness of predictions for such tasks.

## Data Availability

The original contributions presented in the study are included in the article/supplementary material, further inquiries can be directed to the corresponding author/s.
